# Biomimetic, Interface-Free Stiffness-Gradient PDMS-Co-Polyimide-Based Soft Materials for Stretchable Electronics and Soft Robotics

**DOI:** 10.1021/acsmaterialsau.4c00042

**Published:** 2024-11-18

**Authors:** Stephan Schaumüller, Stefan Halama, Peter Prka, Ian Teasdale, Ingrid Graz

**Affiliations:** † Institute of Polymer Chemistry, Johannes Kepler University Linz, 4040 Linz, Austria; ‡ Christian Doppler Laboratory for Soft Structures for Vibration Isolation and Impact Protection (ADAPT), School of Education, STEM Education, Johannes Kepler University Linz, 4040 Linz, Austria

**Keywords:** copolymer, polyimide, polydimethylsiloxane, interface-free gradient, soft materials, soft robotics, stretchable electronics

## Abstract

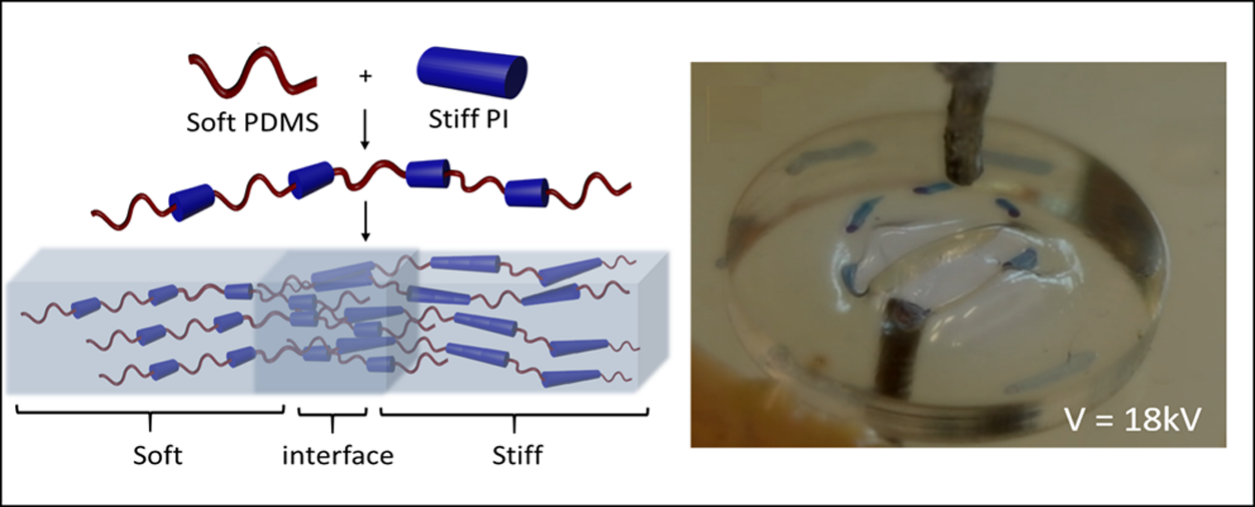

Soft materials play a pivotal role in the efficacy of stretchable electronics and soft robotics, and the interface between the soft devices and rigid counterparts is especially crucial to the overall performance. Herein, we develop polyimide–polydimethylsiloxane (PI–PDMS) copolymers that, in various ratios, combine on a molecular level to give a series of chemically similar materials with an extremely wide Young’s modulus range starting from soft 2 MPa and transitioning to rigid polymers with up to 1500 MPa. Of particular significance is the copolymers’ capacity to prepare seamless stiffness gradients, as evidenced by strain distribution analyses of gradient materials, due to them being unified on a molecular level. The copolymers and gradient materials were successfully used as substrates for stretchable thin-film conductors and tested as dielectric elastomer actuators, demonstrating their potential application as enabling components in stretchable electronics and soft robots.

## Introduction

There is a growing interest in the technical evolution of stretchable electronics^1^ and soft robotics that can mimic nature’s soft materials.^2,3^ Stretchable electronic circuits, inspired by the human skin, undergo extreme deformations while retaining their electrical functionalities such as sensing,^2^ signal processing, and light emission.^4^ Soft robots, inspired by invertebrates, such as squid, move with unlimited degrees of freedom, exploiting novel actuation principles such as pneumatics, phase transitions, and dielectric elastomer actuation (DEA).^5^ Both stretchable electronics and soft robotic devices rely on synthetic soft material platforms such as elastomers, but the use of conventional off-the-shelf materials for engineering stretchable electronics and soft robotics comes with major challenges regarding performance, as well as interfacing them with rigid conventional devices.^6^ Here, the interface between the soft devices and rigid counterparts is especially crucial. Contemporary materials employed in flexible electronics typically combine softness and rigidity to address the requirements of multiple functionalities.^7^ Examples include sensors for health-care applications,^8^ optoelectronic parts,^9^ electronic skin for soft robots,^10^ or flexible analogues of conventional consumer electronics such as mobile phones, e-readers, or smartwatches.^8^ However, the interface between materials with completely different mechanical and chemical properties is an obvious weak point and commonly the site of mechanical failure.^7^ Recently, solutions to this problem are being developed, for example, generating gradients through photopatterning of photoreactive polymers.^11^ Another innovative approach is the development of novel ionogels, with a hydrogen bond toughened polymer-rich phase that dissipates energy and an elastic solvent-rich phase that enables for large strain.^12^

**Figure 1:**
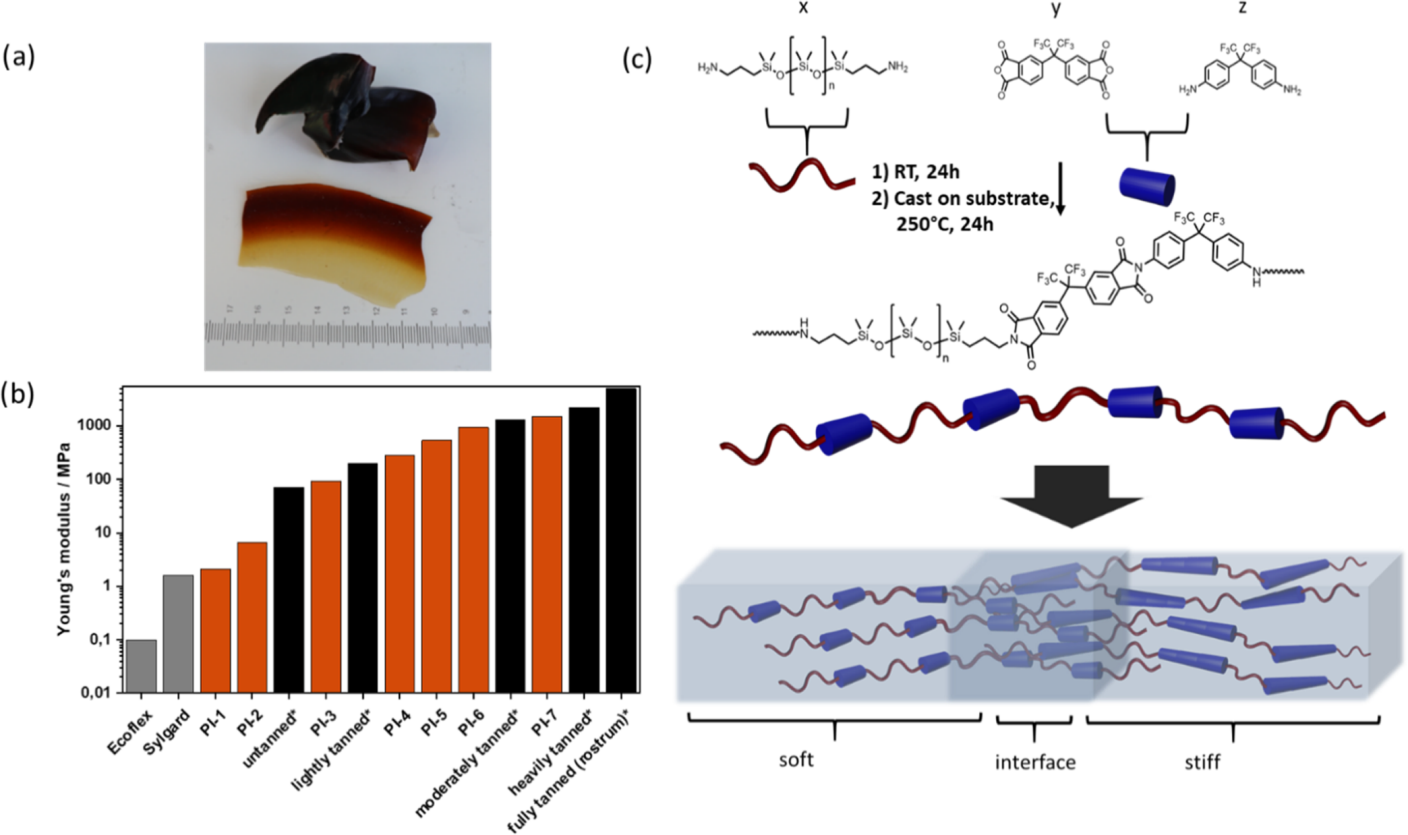
(a) Top: photograph of the beak of a Humboldt squid (*D. gigas*) encompassing a 200-fold modulus gradient. Bottom: a freestanding film of our material showing a continuous gradient. (b) Young’s modulus of commercial PDMS grades compared to our materials (PI 1–7) covering the stiffness range of the squid beak (data for the squid beak taken from ref (13)). (c) Schematic of the synthesized PDMS-polyimide copolymers, whereby (*x* + *z*):*y* = 1:1 and *n* ≈ 10. The chemical similarity and, hence, ideal compatibility of the polymers facilitate the preparation of elastic stiffness-gradient materials (varying PDMS–PI ratios) with a molecularly overlapping, hence mechanically robust interface.

Inspiration can be taken from nature to address this challenge. In nature, soft to rigid transitions are realized by using the same material in a mechanical gradient. A typical example is the beak of a Humboldt squid (*Dosidicus gigas*)^13^ (Figure 1a). Squids are invertebrates capable of squeezing themselves through the tiniest holes due to the extreme deformability of their bodies. However, their beaks are stiff and rigid, allowing the cracking of mussel shells to feed on them. The interface of the rigid beak with the soft body is enabled by a mechanical gradient, as shown in Figure 1. Such a mechanical gradient ensures a gradual transition, limiting the critical strain locally, even when the overall material undergoes deformations that exceed the critical strain for the rigid regions.

Two materials frequently used in this field are polyimide (PI) and polydimethylsiloxane (PDMS). PI foils are well established in electronics as they offer a wide range of beneficial properties such as flame retardancy, flexibility, and insulating properties.^14^ Cross-linked PDMS polymers are established in applications requiring elasticity and softness. A significant challenge, however, is providing high stretchability in direct combination with the long-term stability of electronic components. The active materials can easily peel off from the substrate because of the physical modulus mismatch between the substrate and the active materials.^15^ Another challenge in using PDMS as a substrate for electronics is that the metal and PDMS layers undergo different degrees of thermal expansion.^16^ Chemical surface modification strategies to enhance the adhesion to the active materials are being developed, but they are limited to particular materials like gold and some inorganic semiconductors.^15^

Building on the pioneering work of Clair et al.^17^ and McGrath et al.,^18,19^ we have now developed a copolymer combining the mechanical properties of polyimides (PI) and polydimethylsiloxanes (PDMS) on a molecular level. Systematic variations of these copolymer fractions allow us to customize the mechanical properties of films with a range of Young’s moduli from 2 MPa for soft materials to 1500 MPa for rigid materials. We demonstrate how this approach facilitates the preparation of mechanical gradients within one material, and we also show their potential application in stretchable electronics and soft robotics for the first time.

## Results and Discussion

### Copolymer Synthesis

To prepare soft materials with a broad stiffness range, we combine the complementary properties of polyimides and PDMS on a molecular level. Copolymers were designed in which the stiff polyimide part (blue cylinder) consists of the aromatic anhydride (4,4′-(hexafluoroisopropylidene)diphthalic anhydride) (6FDA) and the aromatic diamine 2,2-bis(4-aminophenyl)hexafluoropropane (BAP6F). These monomers were chosen because of their excellent solubility and the anticipated low chromaticity of the resulting polymers. The flexible PDMS part (red coil, Figure 1c) uses the commercially available aminopropyl-terminated polydimethylsiloxane (DMS-A11) with a dimethylsiloxane repeating number of *n* ≈ 10 (as determined by NMR spectroscopy). To vary the molecular and bulk properties, the DMS-A11 (*x*) was replaced in various ratios with the aromatic diamine (*z*) while maintaining the 1:1 stoichiometry with the dianhydride (*y*) to yield polymers **PI-1** to **PI-7** (Table 1).

**Table 1:** Overview of the Molecular Composition of the Copolymers and Their Material Properties

	*x*	*y*	*z*	gel fraction (%)	Young’s modulus (MPa)	tan δ_β_	*T*_g1_ (°C)	tan δ_α_	*T*_g2_ (°C)
**PI-1**	1	1	0	69.2	2.1	0.15	–89	0.77	22
**PI-2**	0.75	1	0.25	39.5	6.7	0.14	–99	0.71	43
**PI-3**	0.625	1	0.375	42.1	94	0.12	–97	0.57	88
**PI-4**	0.5	1	0.5	17.4	284	0.09	–101	0.57	136
**PI-5**	0.375	1	0.625	45.2	537	0.09	–105	0.67	192
**PI-6**	0.25	1	0.75	50.0	937	0.08	–114	0.86	236
**PI-7**	0	1	1	0	1495			1.06	362

The films of **PI-1** to **PI-7** were then cast from solutions of 50% *N*-methylpyrrolidone (NMP) and 50% tetrahydrofuran (THF) of the corresponding polyamic acid (PAA) precursors before undergoing thermal imidization at 250 °C for 24 h. FT-IR spectroscopy (Figure S1) indicated the complete conversion of amic acid into imide groups in the absence of CO_2_H stretch vibrations. Moreover, increased Si–O stretch vibrations at 1010 cm^–1^ and aliphatic C–H stretch vibrations at 2960 cm^–1^ can be observed with an increasing DMS-A11 (*x*) content. Thermogravimetric analysis (TGA) measurements under nitrogen showed high thermal stability of all polymers, with **PI-1** (the polymer with the highest PDMS content) having a T_95_ of 408 °C. As expected, this value continually increases with decreasing PDMS content to 443 °C in **PI-6**, and the value of **PI-7** is set apart at 497 °C (see Figure S2).

### Thermomechanical Characterization

**Figure 2:**
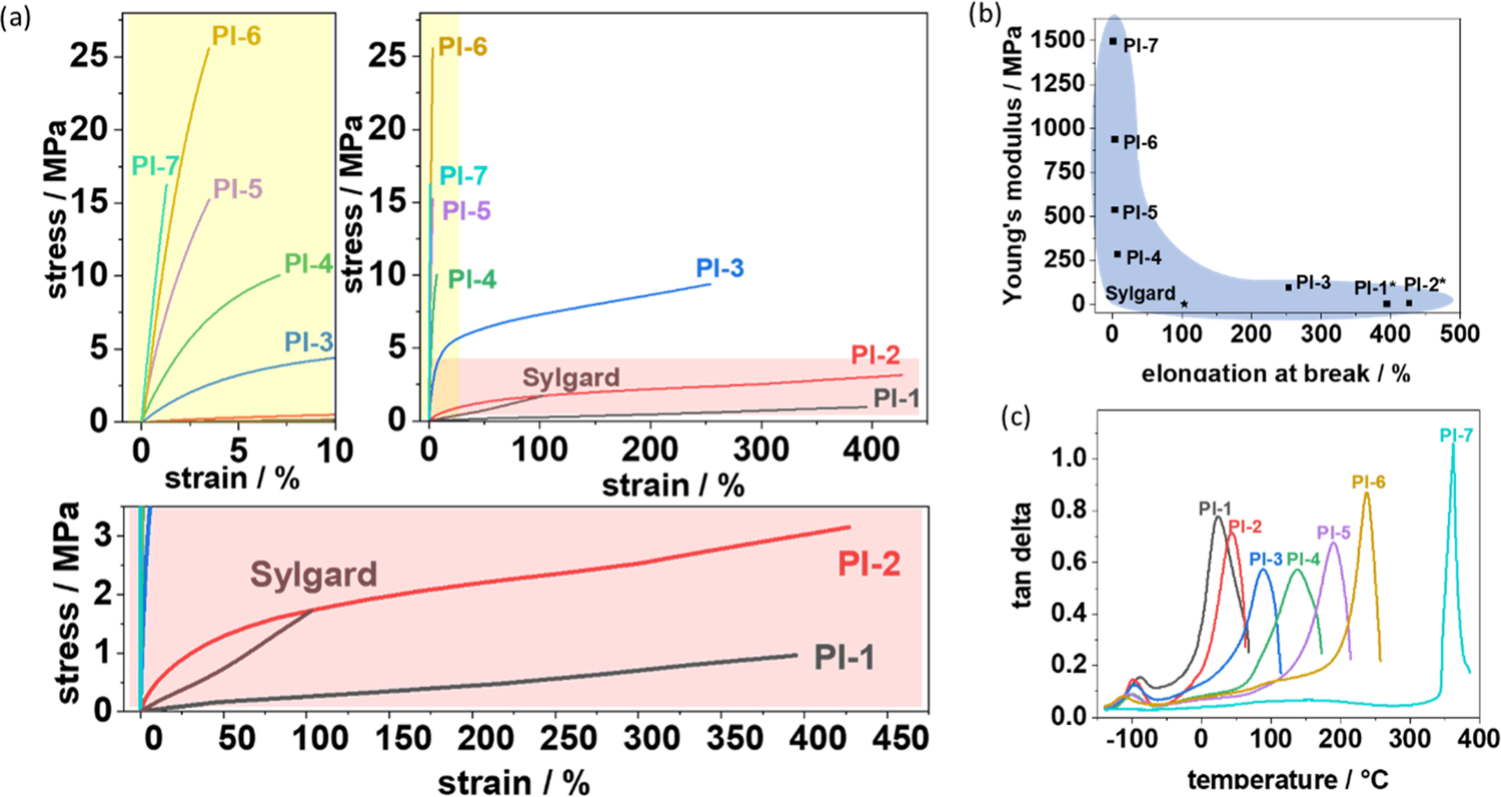
(a) Stress–strain curves and (b) Ashby plot of “Youngs modulus” and “elongation at break of the polymer series”. *PI-1 and **PI-2** did not break during the DMTA measurement but reached the maximum possible length of the testing device. Sylgard 184 was added as a commercial reference material. (c) tan δ curves of the polymer series.

Through simple variations of monomer feed ratios, Young’s modulus of the polymers can be adjusted from as high as 1495 MPa in **PI-7** to as low as 2.1 MPa in **PI-1**. Stress–strain curves of the solution-cast films showed a clear relationship between PDMS content and modulus (Figure 2a) upon increasing the *x*:*z* ratio. This synthesis method therefore represents a simple route to materials covering the stiffness range of the beak of a Humboldt squid (*D. gigas*) with a single chemical procedure. Furthermore, the elongation at break increased dramatically from 1.3% in **PI-7** to >400% for **PI-1** and **PI-2**, due to the hidden length of the flexible, coiled PDMS segments. Indeed, **PI-1** and **PI-2** did not fracture in the dynamic mechanical thermoanalysis (DMTA), but they could not be strained any further because of the physical limitations of the device. It is estimated that **PI-1** can be stretched by approximately 500% based on hand-conducted strain tests. Again, a clear correlation was observed between the PDMS content and elongation at break. A commercial PDMS formulation (Sylgard) is shown for comparison, having similar Young’s modulus as **PI-1** and **PI-2** but considerably lower elongation at break at 103%. An Ashby plot of the mechanical properties (Figure 2b) shows the achievable property range offered by the slight variations in the molecular structure. Polymers with a higher PI fraction display a higher modulus and lower elongation at break due to rigid PIs segments (visualized by blue cylinders in Figure 1c) dominating the mechanical properties of the polymer. Polymers with *x* ≥ 0.6 (**PI-1** to **PI-3**) show high elongation at break and low modulus; hence, the PDMS component (red coils) dominates in the macroscopic behavior of the material.

**Figure 3:**
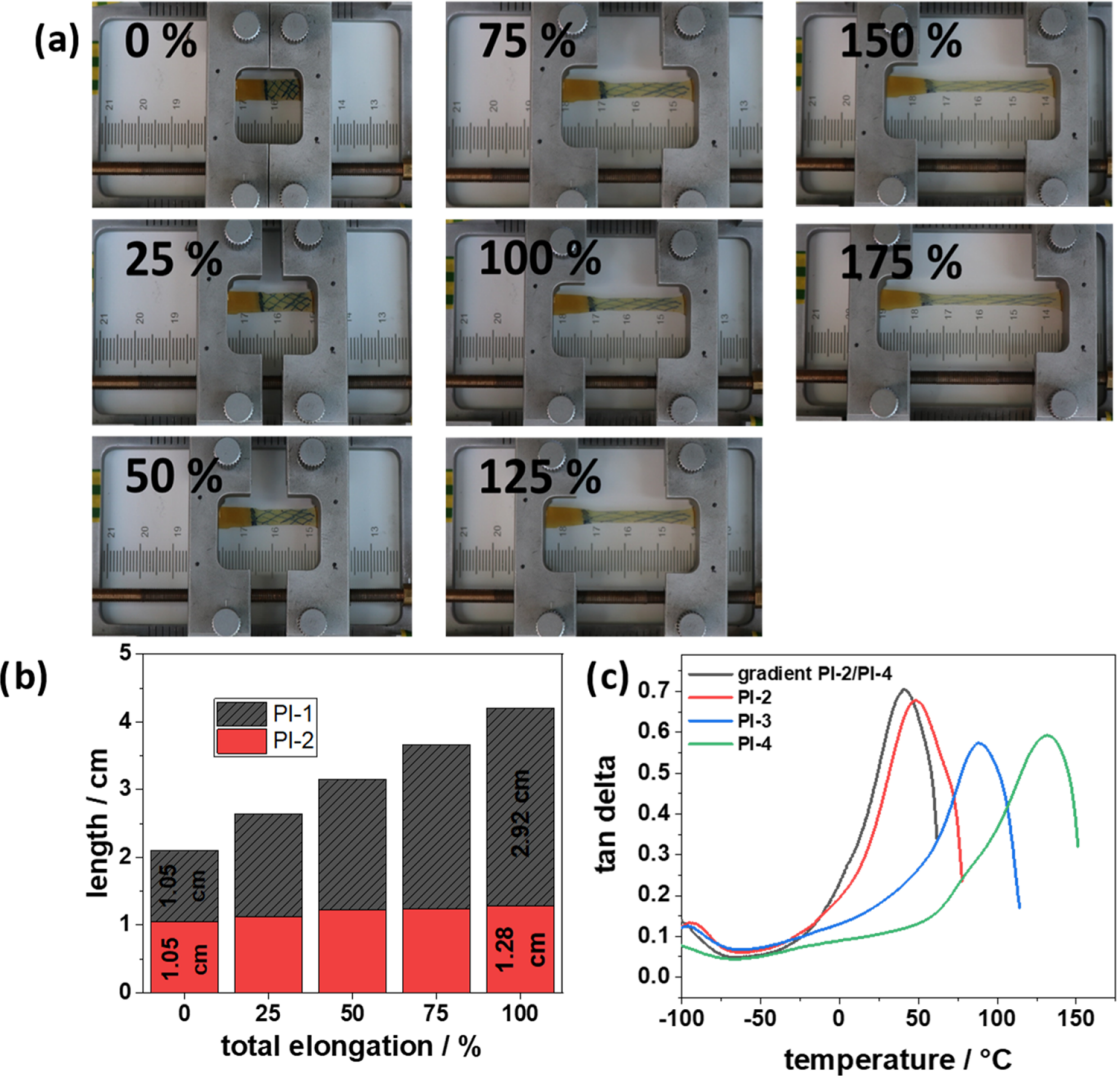
(a) Two-component film made of **PI-1** (checkered, right) and **PI-2** (clear, left) is placed in a stretching device in which the two sides can be displaced and moved apart simultaneously. (b) Graph showing the length of the two parts of the film displayed in (a) at different stages of elongation. (c) DMTA measurements of a two-component film made of **PI-2** and **PI-4**, compared to the neat films as well as **PI-3**.

Interestingly, a clear trend in the tan δ values is observed with varying PDMS content, as measured by DMTA (Figure 3c). Two clear transitions are observed, indicating the microphase separation of the PDMS and PI-phases.^18^ The lower transition *T*_β_ is in the region of −100 °C, as expected for PDMS and increases slightly with the PI content. The upper transition (*T*_α_) shows a clear dependence on PI content and is lowered compared to the pristine **PI-7**, presumably due to shortening PI segment lengths. It was possible to vary the upper softening point of the polymers from 22 to 236 °C for PDMS-containing copolymers (Figure 2c). Overall, all PI–PDMS copolymers exhibit high energy-dissipation properties with a loss factor tan δ larger than 0.5, dependent on their respective composition. Their damping performance spans temperatures from 0 to 350 °C, even up to 400 °C for **PI-7**.

### Interface-Free Gradient Polymers

Due to the similar chemical nature but different mechanical properties of the copolymer films, we investigated the polymers as continuous gradient materials. To this end, we cast polymers of different moduli next to each other before subjecting them to a thermal imidization temperature schedule. A two-component film consisting of **PI-1** and **PI-2** is placed in a stretching device in which the two sides can be displaced and moved apart simultaneously. Upon stretching, the soft part is elongated from 1.05 to 4.57 cm, whereas the hard part is only stretched from 1.05 to 1.61 cm. The gradient polymers show no tearing at the interface, and hence, this continuous gradient material can potentially overcome the problematic interface between hard and soft materials. Indeed, this behavior can also be observed in the DMTA scans of the polymer gradient series: another gradient film (composed of **PI-2** plus **PI-4**) was tested in the DMTA, showing behavior very similar to the softer component (**PI-2**) (Figure 3c). The tan δ curve of **PI-3** is shown for comparison, as this has the same total ratio of aromatic diamine to PDMS-diamine as the gradient material. In this case, the properties lie, as expected, between those of **PI-2** and **PI-4**.

### Creep Measurements

**Figure 4:**
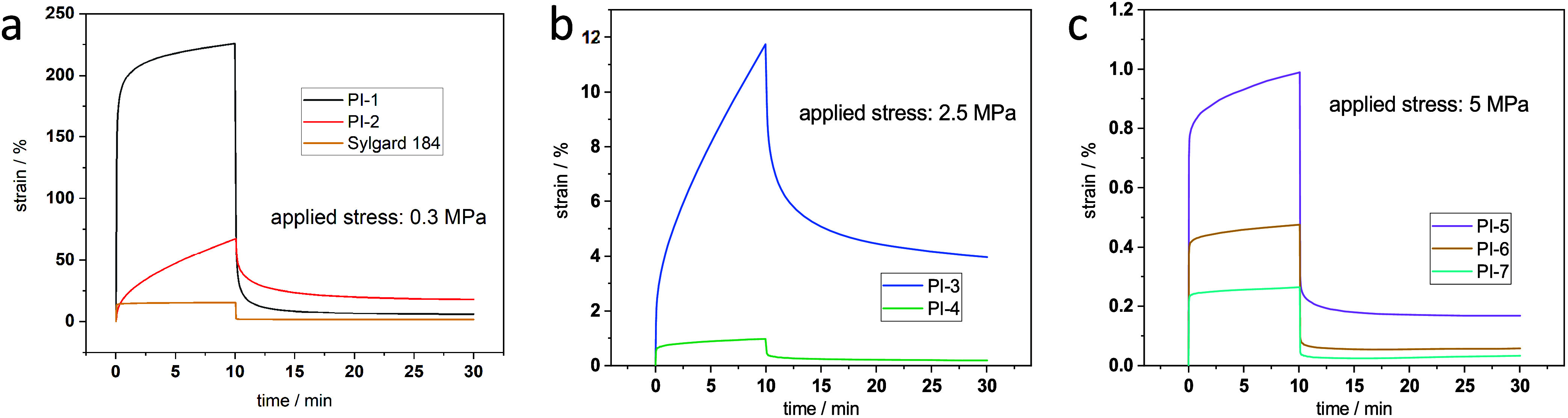
Creep measurements: The applied stress varied for the different polymers. (a) **PI-1**, **PI-2**, and Slygard 184 (for reference): 0.3 MPa. (b) **PI-3** and **PI-4**: 2.5 MPa. (c) **PI-5**, **PI-6**, and **PI-7**: 5 MPa.

Creep studies were conducted to investigate the mechanical behavior further. The samples were put under a defined stress for 10 min and then relaxed for 20 min. However, it needs to be said that the polymers were not all exposed to the same stress during the creep tests, which was not possible due to the considerably different mechanical properties of the polymers. In Figure 4a–c, the creep measurements are grouped according to applied stress. It is visible that all samples except for **PI-2** and **PI-3** show linear viscoelastic behavior. Linear viscoelastic behavior is characterized by strain increasing instantaneously when constant force is applied in the elastic response. Further, when the force is maintained, the strain continuously increases, however, at a slower rate. This creep represents a delayed but slower increase of the strain characterizing the viscoelastic response. Once the force is removed, the recovery stage starts with a steep elastic decline in strain, followed by a slower viscous decrease, implying the presence of a viscoelastic region with a potential permanent deformation. To be precise, the deformation is not permanent but rather represents a delayed process with a very large time frame that exceeds the 10 min period after unloading. Summarizing Figure 4, **PI-1** and **PI-2**, the softest copolymers were subjected to the testing procedure with a force of 0.3 MPa. The data shown in Figure 4a shows that the elastic response is around 180% with the viscoelastic response of 5.8% for **PI-1** and approximately 10% with 18% for **PI-2**.^20^**PI-3** and **PI-4** display linear viscoelastic behavior with about 2% elastic response and about 4% viscoelastic response and less than 0.6 and 0.2%, respectively.

### Dielectric Spectroscopy

**Figure 5:**
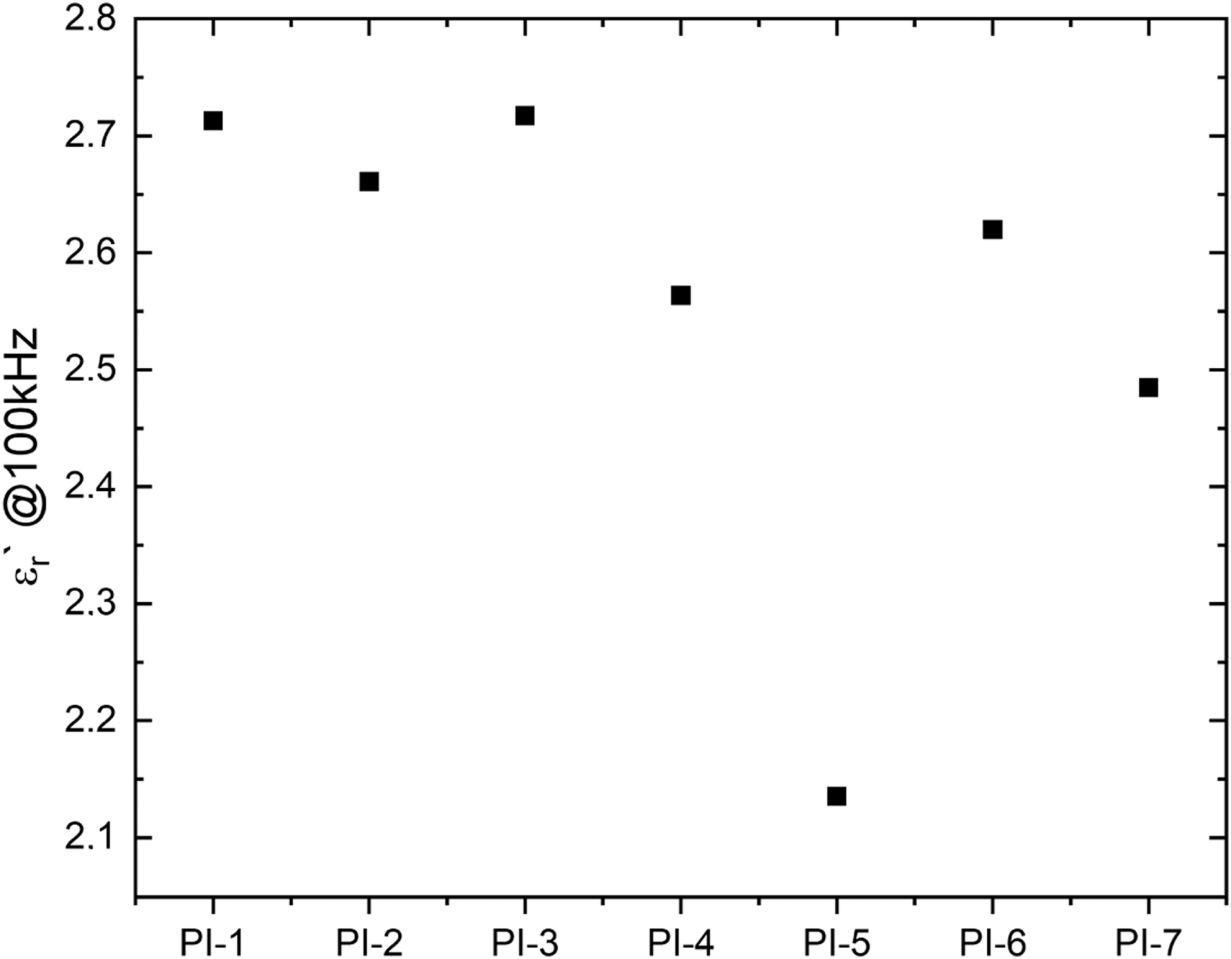
Dielectric constant ε_r_′ measured at 100 kHz for decreasing PDMS content.

Dielectric constant ε_r_′ and dissipation factor ε_r_″ were evaluated by means of dielectric spectroscopy using an E4990A impedance analyzer with a 16451B Dielectric Test Fixture (both Keysight) with electrode B in contact mode. Cast PI–PDMS films with thicknesses between 180 and 310 μm prepared as described above were clamped within the fixture and measured between 100 Hz and 100 kHz. Sylgard 184 in a 10:1 mixing ratio exhibits a dielectric constant of 2.68@100 kHz.^21^ The dielectric constants (and dissipation factors) @100 kHz, from highest to lowest PDMS content, range from 2.7 to 2.5. All data are summarized in Table 2. With the exception of **PI-5**, where the sample contained air bubbles, the dielectric constant is slightly decreasing with decreasing PDMS content as shown in Figure 5. Due to the varying thickness of the samples itself, the estimated error is approximately 5%. Detailed dielectric measurement data for each copolymer can be found in the Supporting Information (Figures S16–S22).

**Table 2:** Overview of Dielectric Constants εr′ and Dissipation Factors εr″ of the Prepared Copolymers

	ε_r_′ (@100 kHz)	ε_r_″(@100 kHz)
**PI-1**	2.71271	0.03512
**PI-2**	2.66068	0.01554
**PI-3**	2.71719	0.01257
**PI-4**	2.56352	0.00694
**PI-5**	2.13531	0.00359
**PI-6**	2.61972	0.00602
**PI-7**	2.48471	0.00579

## Applications

### Stretchable Electrodes

**Figure 6:**
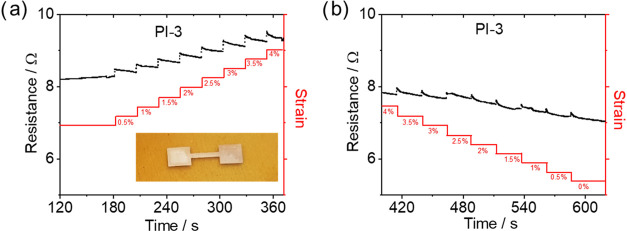
Resistance versus time behavior is shown with a stepwise increase of resistance upon each stretching step and relaxing upon release of the strain. A stretchable conductor on a **PI-3** sample (a) stretched up to 4% strain and (b) relaxed. Inset photo: stretchable conductor on **PI-3**.

Given their mechanical properties, the PI–PDMS copolymers, are highly interesting as a platform for stretchable electronics.^22^ Especially, the potential of creating mechanical gradients, as shown in Figure 3, would enable more reliable transitions from rigid to stretchable electronics.^23^ Interestingly, PI is often the material of choice in stretchable electronic circuits^24,25^ particularly for rigid islands, facilitating such transitions that warrant electrical functionalities. Selected copolymers were tested for their applicability as thin-film stretchable electrodes. Here, thin-metal layers of 5 nm chromium and 50 nm gold are directly deposited by thermal evaporation. Their low thickness, together with the mechanical properties of the substrate, causes the latter’s mechanics to prevail. This enables these thin-metal-film electrodes to be reversibly stretched, exceeding the rupture strain of bulk gold. Samples were stretched uniaxially well below their elastic limit, determined by creep measurements (Figure 4). Figure 6 shows the resistance versus time behavior of a **PI-3** sample subjected to a loading cycle in 0.5% steps with 30 s resting periods in between up to a maximum of 4% and an unloading cycle.

The resistance increases stepwise upon loading and decreases upon the release of the strain. After the stretching cycle, the resistance of the relaxed conductor is even lower than the initial resistance. This electromechanical behavior is typical for thin-film stretchable conductors,^26^ where the as-prepared films exhibit microcracks upon thermal expansion of the substrate. These microcracks open up during uniaxial stretching, releasing strain that could result in rupture and increasing the percolation path, resulting in a stepwise increase in the resistance with strain. **PI-1** and **PI-2** samples were also tested for their usability as stretchable conductors. Both samples remain conductive during stretching, again showing a stepwise increase and decrease of resistance as a function of strain. Figure S12 shows the resistance versus strain data of conductors on **PI-1** and **PI-2** samples with a maximal uniaxial strain of 27 and 10% upon loading. The conductors were stretched stepwise in 2.7% steps with 60 s rest time and 1% steps, respectively, with a 10 s rest time. These results, therefore, demonstrate the potential applicability of PI–PDMS for stretchable electrodes. The findings are very much similar to identically prepared 5 nm chromium/50 nm gold conductors using Sylgard 184 as a stretchable substrate. When stretched up to 20% uniaxially, resistance increases stepwise with strain and upon releasing returns to its initial value.^27^ In comparison, the 4% demonstrated with **PI-3** seem small; however, **PI-1** and **PI-2** samples were also tested for their usability as stretchable conductors (Video S1).

### Dielectric Elastomer Actuators

After a successful demonstration of the usability of PI–PDMS for stretchable electrodes, their use in soft robotic devices was investigated. Here, we studied their applicability as dielectric elastomer actuators (DEAs).^28^ Dielectric elastomer actuators, in their most simple form, use a thin elastomer film mounted on a frame. When an electric voltage is applied by means of needle electrodes brought in close proximity of either side of the elastomer, the opposite charges on both electrodes attract each other due to the Coulomb force, resulting in electrostatic pressure,^29^ which then compresses the elastomer in the *z*-direction (thickness). At the same time, the charges of the same sign within each stretchable electrode repel each other, stretching the elastomer in the plane. Because of the incompressibility of elastomers (with a Poisson ratio of 0.49), the elastomer will conserve its volume and thus expand in area.

**Figure 7:**
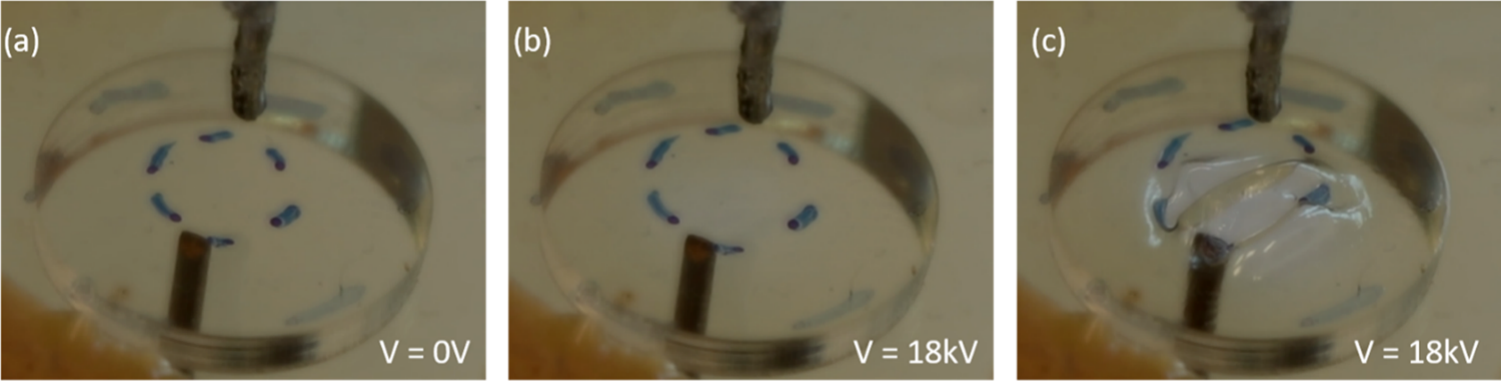
Dielectric elastomer actuator under actuation. (a) Elastomer membrane prestretched to 135% mounted at no voltage applied. (b) Upon applying a high voltage, the membrane starts to actuate around 18 kV and (c) is constantly expanding and thereby thinning out. When the area expansion exceeds 100%, wrinkling is observed.

Figure 7 shows a square sample of **PI-1** prestretched to 135% and mounted on a poly(methyl methacrylate) (PMMA) frame with a circular hole to obtain a freestanding membrane. The experimental setup is shown in Figure S13. When a rectangular signal of 18 kV was applied to the membrane by two needle electrodes, the spray-on charges clearly actuated the copolymer by expanding its area by 30%. This is visible in Figure 7b in an expansion of the blue dashed ring. Subsequently, the area in proximity to the needles locally expands even further, while the charges thin the copolymer to such an extent that it starts to first wrinkle and then sag with an area expansion that exceeds 100% (Video S1). When using two-component (PI-1 and PI-3, PI-1 and PI-2) films in the DEA setup, actuation primarily occurs in the softer component (Figure S14). This enables the design of directional DE actuators. Overall, these results show that copolymers can be employed for DEAs and that it is on par with the performance of silicones such as NuSil CF19-2186.^30^

## Conclusions

A series of PI–PDMS copolymers have been synthesized and characterized, thus combining on a molecular level the distinctive attributes of silicones, typified by high elasticity, and the elevated rigidity characteristic of polyimides. These novel copolymers offer a highly modifiable platform concerning mechanical attributes, displaying pronounced damping characteristics alongside the capacity for gradient formation within a singular material matrix, thereby positioning them as promising contenders within the realm of soft electronics. The efficacy of stretchable thin-film conductors deposited onto PI–PDMS copolymers has been validated, exhibiting a concomitant increase in electrical resistance proportional to applied strain, indicative of their utility as strain-sensing elements. Additionally, the functionality of PI–PDMS copolymers as dielectric elastomer actuators has been demonstrated, underscoring their prospective integration within soft robotic systems. The capacity for gradient formation assumes particular significance within both stretchable electronics and soft robotics domains, as exemplified by the differential strain distributions observed across graded samples and the consequent actuation behaviors. With optimization of the casting techniques and more precise control over film thickness, further enhancement of the material performance is foreseeable. Thus, the developed PI–PDMS copolymers exhibit substantial potential across diverse applications within soft electronics.

## Experimental Section

### Materials and Methods

Unless stated otherwise, all starting materials and solvents were purchased in reagent grade. 6FDA from TCI was dried at 200 °C before use, BAP6F was obtained from TCI, and DMS-A11 (*M*_n_ 850–900 Da, *n* ∼ 10) was purchased from Gelest. Tetrahydrofuran (THF, 100% Normapur from VWR), *N*-methylpyrrolidone (NMP, 99.8% peptide grade), and acetic anhydride (from Merck) were used as received. Triethylamine from Merck was freshly distilled before use.

For mechanical testing, a DMA Q800 instrument from TA Instruments equipped with a liquid nitrogen GCA was used. Thermogravimetric analysis (TGA) curves were recorded on a TA Instruments Q5000 under nitrogen. IR (infrared) spectra were measured on a PerkinElmer Spectrum 100 FT-IR spectrometer equipped with an ATR using a scan number of 4. The ^1^H nuclear magnetic resonance (NMR) spectra were recorded on a Bruker Avance III 300 MHz spectrometer. For determination of gel fractions, polymer films with approximately 100 mg were weighed, immersed in dichloromethane for 2 × 24 h, and weighed again after drying.

### Chemical Synthesis

The formation of polyamic acid (PAA) is carried out in a 1:1 volumetric mixture of NMP and THF.^18^ An example of the synthesis of PI-2 is given. To 0.7503 g (0.75 mmol) of DMS-A11 in a round-bottom flask were added 0.4442 g (1 mmol) of 6FDA. Five mL of NMP and THF were added, and the resulting light yellow solution was stirred at room temperature for 30 min. Subsequently, 0.0835 g (0.25 mmol) of BAP6F were added. The mixture was stirred at room temperature for another 24 h to yield a viscous, clear, light yellow solution.

The PAA solution was cast onto poly(tetrafluoroethylene) (PTFE) sheets and put into a drying cabinet with the following heating schedule: 30 min at 100 °C, 30 min at 150 °C, 30 min at 200 °C, and 12 h at 250 °C. The fully formed PI sheets can be peeled off the PTFE substrates after cooling to RT.

### Thermomechanical Characterization

Measurements were conducted in a tensile configuration using dynamic mechanical analysis (DMA Q800 V21.3, TA Instruments). All data was recorded from rectangular film samples with 10.60 mm × 5.00 mm and a thickness of approximately 0.12 mm. Stress–strain curves were recorded at 30 °C with a force ramp of 3 N/min and a maximum force of 18 N. Tan δ measurements were conducted with a fixed applied force of 0.1 N starting from −140 °C at a rate of 1 Hz. Creep measurements were conducted with a fixed applied force for 10 min and a 20 min recovery period with no applied force at a constant temperature of 30 °C and ambient pressure. The applied force was adjusted depending on the PDMS content to avoid damaging/rupturing the samples as follows: **PI-1** and **PI-2**: 0.3 MPa, **PI-3** and **PI-4**: 2.5 MPa. (c) **PI-5**, **PI-6** and **PI-7**: 5 MPa.

### Applications

#### Stretchable Electrodes

Stretchable conductors were prepared by means of thermal evaporation (Pfeiffer Balzer PLS570) of 5 nm chromium and 50 nm of gold successively onto **PI-1**, **PI-2**, and **PI-3** samples. Patterning of the 1 mm wide and 5 mm long conductors with 5 × 5 mm^2^ conducting pads was achieved by shadow masking. The stretchable conductor was evaluated using a custom-built computer-controlled tensile testing setup and a multimeter (DMM7510, Keithley) set to read 6 data points per second. Electrical contact was achieved by means of thin copper wires and Epotec H27D conductive paste part B.

#### Dielectric Elastomer Actuators

Square samples of copolymers were prestretched and mounted on a poly(methyl methacrylate) (PMMA) frame with a 20 mm diameter circular hole in its center. Good adhesion is solely granted here by the stickiness of the copolymer samples. Needle electrodes were positioned in close proximity, and a rectangular high-voltage signal was applied by means of a function generator (DG1022, Rigol) amplified by a high-voltage amplifier (Trek 20/20C-HS ±, Advanced Energy). Evaluation of actuation was performed by video recording and ImageJ.

## Supporting Information Available

FT-IR spectra, TGA data, DMTA data, 1H NMR spectra, data for dielectric constants, and dissipation factor measurements and additional experiments for the stretchable electronics and DEA demonstrations (PDF)
A video showing the DEA behavior of PI-1 and a document with the following additional experimental data (Video S1) (MP4)

## References

[ref1] Kim D. C.; Shim H. J.; Lee W.; Koo J. H.; Kim D. Material-Based Approaches for the Fabrication of Stretchable Electronics. Adv. Mater. 2020, 32, 1902743 10.1002/adma.201902743. 31408223

[ref2] Bauer S.; Bauer-gogonea S.; Graz I.; Kaltenbrunner M.; Keplinger C.; Schwödiauer R. 25th Anniversary Article : A Soft Future : From Robots and Sensor Skin to Energy Harvesters. Adv. Mater. 2014, 26, 149–162. 10.1002/adma.201303349. 24307641 PMC4240516

[ref3] Whitesides G. M. Soft Robotics. Angew. Chem., Int. Ed. 2018, 57, 4258–4273. 10.1002/anie.201800907. 29517838

[ref4] White M. S.; Kaltenbrunner M.; Głowacki E. D.; Gutnichenko K.; Kettlgruber G.; Graz I.; Aazou S.; Ulbricht C.; Egbe D. A. M.; Miron M. C.; Major Z.; Scharber M. C.; Sekitani T.; Someya T.; Bauer S.; Sariciftci N. S. Ultrathin, Highly Flexible and Stretchable PLEDs. Nat. Photonics 2013, 7, 811–816. 10.1038/nphoton.2013.188.

[ref5] El-atab N.; Mishra R. B.; Al-modaf F.; Joharji L.; Alsharif A. A.; Alamoudi H.; Diaz M.; Qaiser N.; Hussain M. M. Soft Actuators for Soft Robotic Applications : A Review. Adv. Intell. Syst. 2020, 2, 2000128 10.1002/aisy.202000128.

[ref6] Majidi C. Soft-Matter Engineering for Soft Robotics. Adv. Mater. Technol 2019, 4, 1800477 10.1002/admt.201800477.

[ref7] Wei H.; Mizuki T. W.; Siqian W.; Yasuyuki N.; Ikumu W.; Naito M. Postprogrammable Network Topology with Broad Gradients of Mechanical Properties for Reliable Polymer Material Engineering. Chem. Mater. 2021, 33 (17), 6876–6884. 10.1021/acs.chemmater.1c01715.

[ref8] Harris K. D.; Elias A. L.; Chung H. J. Flexible Electronics under Strain: A Review of Mechanical Characterization and Durability Enhancement Strategies. J. Mater. Sci. 2016, 51 (6), 2771–2805. 10.1007/s10853-015-9643-3.

[ref9] Alim M. D.; Glugla D. J.; Mavila S.; Wang C.; Nystrom P. D.; Sullivan A. C.; Mcleod R. R.; Bowman C. N. High Dynamic Range (Δ n) Two-Stage Photopolymers via Enhanced Solubility of a High Refractive Index Acrylate Writing Monomer. ACS Appl. Mater. Interfaces 2018, 10 (1), 1217 10.1021/acsami.7b15063. 29235344

[ref10] Ha M.; Lim S.; Cho S.; Lee Y.; Na S.; Baig C.; Ko H. Skin-Inspired Hierarchical Polymer Architectures with Gradient Sti Ff Ness for Spacer-Free, Ultrathin, and Highly Sensitive Triboelectric Sensors. ACS Nano 2018, 12 (4), 3964 10.1021/acsnano.8b01557. 29620871

[ref11] Huang S.; Steven M.; Pichumani P. S.; Wallin T. J.; et al. One-Pot Ternary Sequential Reactions for Photopatterned Gradient Multimaterials. Matter 2023, 6 (6), 2419–2438. 10.1016/j.matt.2023.05.040.

[ref12] Wang M.; Zhang P.; Shamsi M.; Thelen J. L.; Qian W.; Truong V. K.; Ma J.; Hu J.; Dickey M. D. Tough and Stretchable Ionogels by in Situ Phase Separation. Nat. Mater. 2022, 21, 359–366. 10.1038/s41563-022-01195-4. 35190655

[ref13] Miserez A.; Schneberk T.; Sun C.; Zok F. W.; Waite J. H. The Transition from Stiff to Compliant Materials in Squid Beaks. Science 2008, 319 (5871), 1816–1819. 10.1126/science.1154117. 18369144 PMC2754134

[ref14] Lu Q.; Zheng F. Polyimides for Electronic Applications. In Advanced Polyimide Materials; Elsevier Inc., 2018; Chapter 5. 10.1016/B978-0-12-812640-0.00005-6.

[ref15] Qi D.; Zhang K.; Tian G.; Jiang B.; Huang Y. Stretchable Electronics Based on PDMS Substrates. Adv. Mater. 2021, 33 (6), 1–25. 10.1002/adma.202003155. 32830370

[ref16] Schmid H.; Wolf H.; Allenspach R.; Riel H.; Karg S.; Michel B.; Delamarche E. Preparation of Metallic Films on Elastomeric Stamps and Their Application for Contact Processing and Contact Printing. Adv. Funct. Mater. 2003, 13 (2), 145–153. 10.1002/adfm.200390021.

[ref17] Maudgal S.; Clair T. L. S. Preparation and Characterization of Siloxane- Containing Thermoplastic Polyimides. Int. J. Adhes. Adhes. 1984, 4 (2), 87–90. 10.1016/0143-7496(84)90105-2.

[ref18] McGrath J. E.; Dunson D. L.; Mecham S. J.; Hedrick J. L. Synthesis and Characterization of Segmented Polyimide-Polyorganosiloxanecopolymers. Adv. Polym. Sci. 1999, 140, 62–105. 10.1007/3-540-49815-x_3.

[ref19] Arnold C. A.; Summers J. D.; Chen Y. P.; Bott R. H.; Chen D.; McGrath J. E. Structure-Property Behaviour of Soluble Polyimide–Polydimethylsiloxane Segmented Copolymers. Polymer 1989, 30, 986–995. 10.1016/0032-3861(89)90068-2.

[ref20] Pascual-francisco J. B.; Farfan-cabrera L. I.; Susarrey-huerta O. Characterization of Tension Set Behavior of a Silicone Rubber at Different Loads and Temperatures via Digital Image Correlation. Polym. Test. 2020, 81, 106226 10.1016/j.polymertesting.2019.106226.

[ref21] Dow. SYLGARD 184 Silicone Elastomer Technical Data Sheet, 2017. https://www.dow.com/en-us/document-viewer.html?docPath=/content/dam/dcc/documents/11/11-3184-01-sylgard-184-elastomer.pdf.

[ref22] Hammock M. L.; Chortos A.; Tee B. C.; Tok J. B.; Bao Z. 25th Anniversary Article : The Evolution of Electronic Skin (E-Skin): A Brief History, Design Considerations, and Recent Progress. Adv. Mater. 2013, 25, 5997–6038. 10.1002/adma.201302240. 24151185

[ref23] Cai D. K.; Neyer A. Realization of Kapton Based Optical Interconnect by KMnO4 Wet Etching. Appl. Phys. A 2010, 99 (4), 783–789. 10.1007/s00339-010-5723-z.

[ref24] Graz I. M.; Cotton D. P. J.; Robinson A.; Lacour S. P. Silicone Substrate with in Situ Relief for Stretchable Thin-Film Transistors Silicone Substrate with in Situ Strain Relief for Stretchable Thin-Film Transistors. Appl. Phys. Lett. 2011, 98 (12), 124101 10.1063/1.3570661.

[ref25] Kim D.-H.; Ahn J.-H.; Choi W. M.; Kim H.-S.; Kim T.-H.; Song J.; Huang Y. Y.; Liu Z.; Chun L.; Rogers J. A. Stretchable and Foldable Silicon Integrated Circuits. Science 2008, 320, 507–511. 10.1126/science.1154367. 18369106

[ref26] Park C.; Lee B.; Kim J.; Lee H.; Ban J.; Song C.; Cho S. J.; et al. Flexible Sensory Systems : Structural Approaches. Polymers 2022, 14 (6), 1232 10.3390/polym14061232. 35335562 PMC8955130

[ref27] Graz I. M.; Cotton D. P. J.; Lacour S. P. Extended Cyclic Uniaxial Loading of Stretchable Gold Thin-Films on Elastomeric Substrates. Appl. Phys. Lett. 2009, 94 (7), 071902 10.1063/1.3076103.

[ref28] Hu P.; Albuquerque F. B.; Madsen J.; Skov A. L. Highly Stretchable Silicone Elastomer Applied in Soft Actuators. Macromol. Rapid Commun. 2022, 43, 2003139 10.1002/marc.202100732. 35083804

[ref29] Keplinger C.; Kaltenbrunner M.; Arnold N.; Bauer S. Röntgen’s Electrode-Free Elastomer Actuators without Electromechanical Pull-in Instability. Proc. Natl. Acad. Sci. U.S.A. 2010, 107 (10), 4505–4510. 10.1073/pnas.0913461107. 20173097 PMC2825178

[ref30] Pelrine R.; Burchfiel B. C.; Lipman P. W.; Parsons T.; Soc G.; Bull A.; Tovish A.; Schubert G.; Luyendyk B. P.; Pelrine R.; Kornbluh R.; Pei Q.; Joseph J. High-Speed Electrically Actuated Elastomers with Strain Greater Than 100%. Science 2000, 287, 836–839. 10.1126/science.287.5454.836. 10657293

