# Supplementary material for: Biomimetic, Interface-Free Stiffness-Gradient PDMS-Co-Polyimide-Based Soft Materials for Stretchable Electronics and Soft Robotics
Source: ACS Mater Au. 2024 Nov 18;5(1):141–8. doi: 10.1021/acsmaterialsau.4c00042 (PMC11718538; doi:10.1021/acsmaterialsau.4c00042)
Supplement: Supplementary file 1 — mg4c00042_si_001.pdf [file mg4c00042_si_001.pdf]

Supporting Information

Biomimetic, interface-free stiffness-gradient PDMS-co-polyimide-based soft materials for stretchable electronics and soft robotics

Stephan Schaumüller, Stefan Halama, Peter Prka, Ian Teasdale and Ingrid Graz**

Stephan Schaumüller - Institute of Polymer Chemistry, Johannes Kepler University Linz, 4040 Linz, Austria; https://orcid.org/0000-0002-5978-4263

Stefan Halama − Christian Doppler Laboratory for Soft Structures for Vibration Isolation and Impact Protection (ADAPT), School of Education, STEM Education, Johannes Kepler University Linz, 4040 Linz, Austria

Peter Prka − Institute of Polymer Chemistry, Johannes Kepler University Linz, 4040 Linz, Austria

Ian Teasdale − Institute of Polymer Chemistry, Johannes Kepler University Linz, 4040 Linz, Austria; orcid.org/664 0000-0001-5953-9084; Email: ian.teasdale@jku.at

Ingrid Graz − Institute of Polymer Chemistry and Christian Doppler Laboratory for Soft Structures for Vibration Isolation and Impact Protection (ADAPT), School of Education, STEM Education, Johannes Kepler University Linz, 4040 Linz, Austria; http://orcid.org/0000-0001-7205-4161, Email: Ingrid.graz@jku.at

CONTENT

FT-IR 2
TGA 3
DMTA 4
Individual storage modulus and loss modulus curves, determined by DMTA 5
Stretchable electrodes 9
Dielectric elastomer actuator sample preparation test 10
NMR 12
Dielectric measurements 13

FT-IR spectra

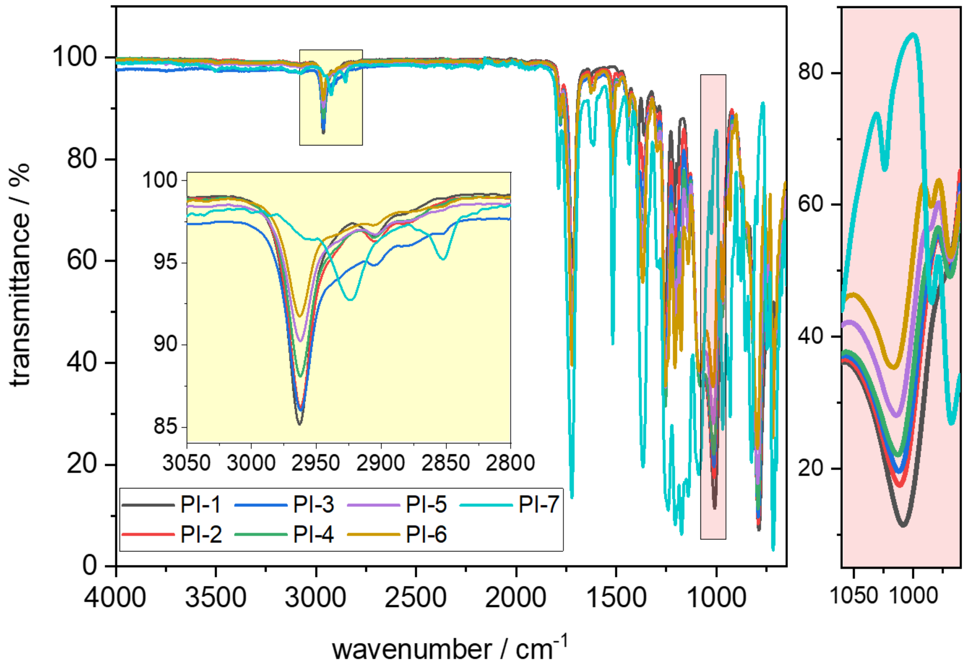

Figure S 1: FT-IR spectra of PI-1 to PI-7.

TGA curves

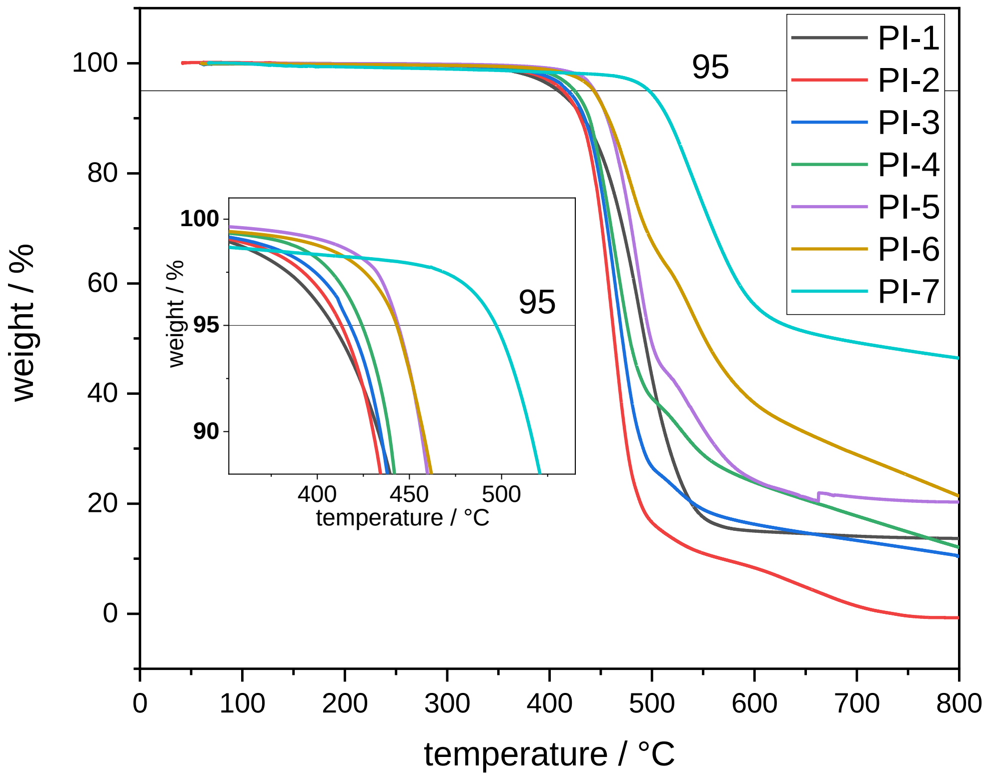

Figure S 2: TGA curves of the polymers, recorded with a heating rate of 10 °C/min.

Polymer	T_{95} / °C
PI-1	408
PI-2	413
PI-3	418
PI-4	424
PI-5	443
PI-6	443
PI-7	497

Table S 1: T95 values determined in the TGA.

DMTA

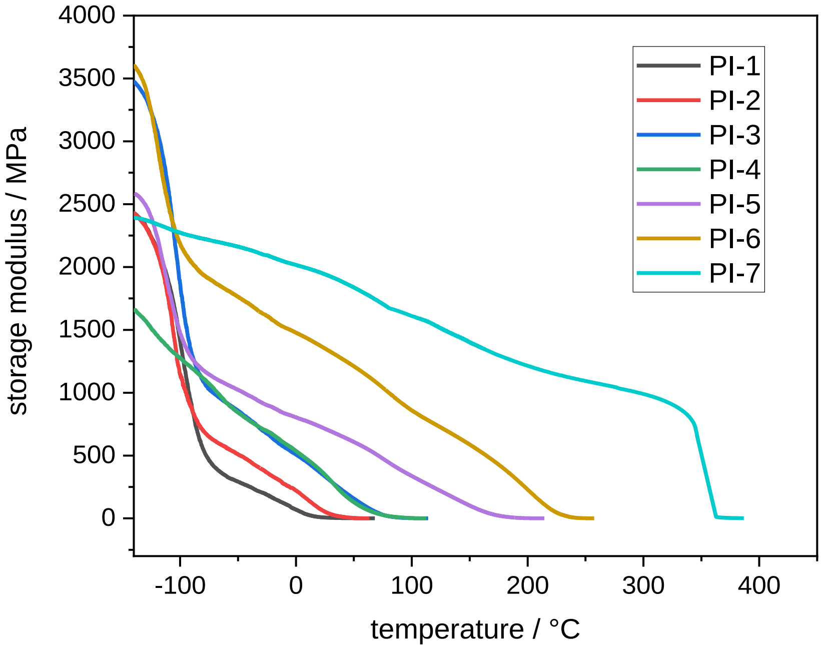

Figure S 3: Comparison of all storage moduli.

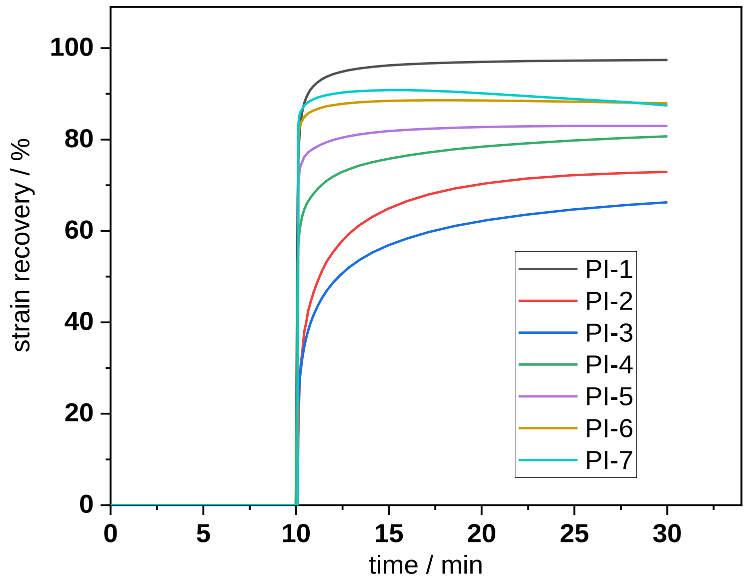

Figure S 4: Percentual strain recovery of the polymers over time. The applied stress varied for the different polymers. PI-1 and PI-2: 0.3 MPa. PI-3 and PI-4: 2.5 MPa. PI-5, PI-6 and PI-7: 5 MPa.

Individual storage modulus and loss modulus curves, determined by DMTA

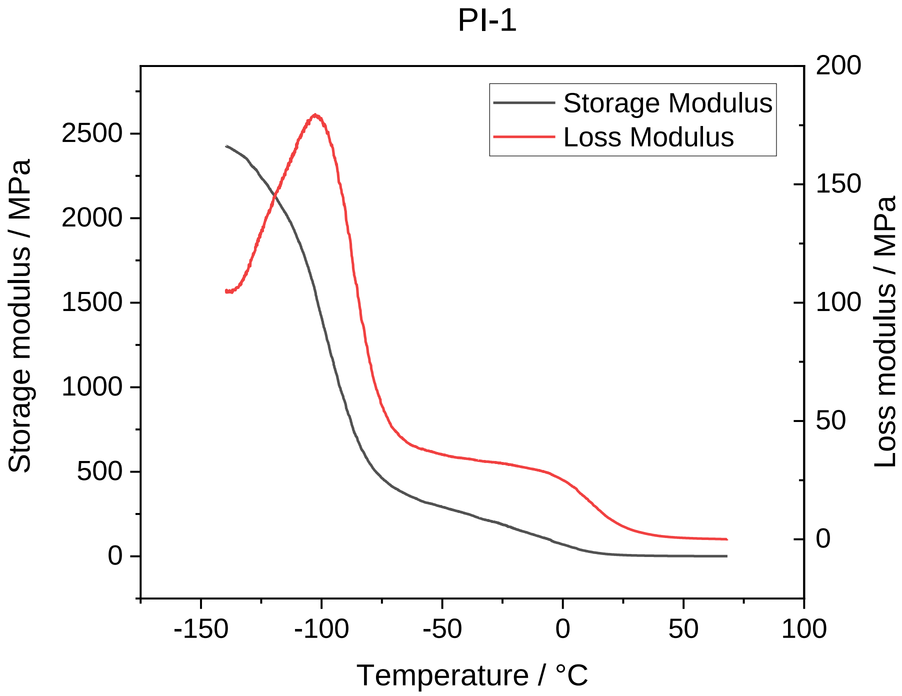

*Figure S 5: Storage and loss modulus curves of **PI-1.***

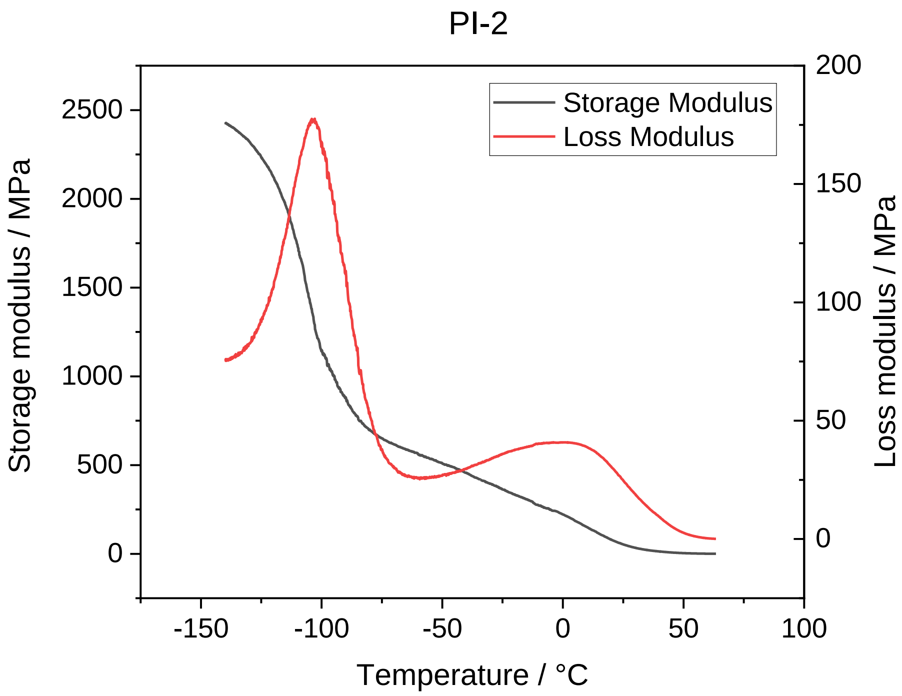

*Figure S 6: Storage and loss modulus curves of **PI-2.***

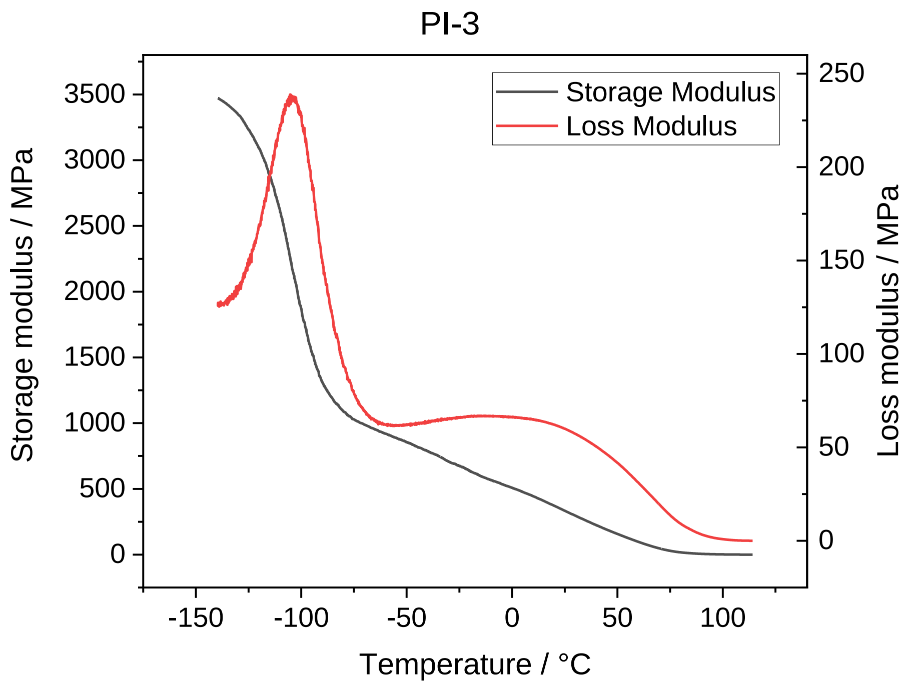

Figure S 7: Storage and loss modulus curves of ***PI-3.***

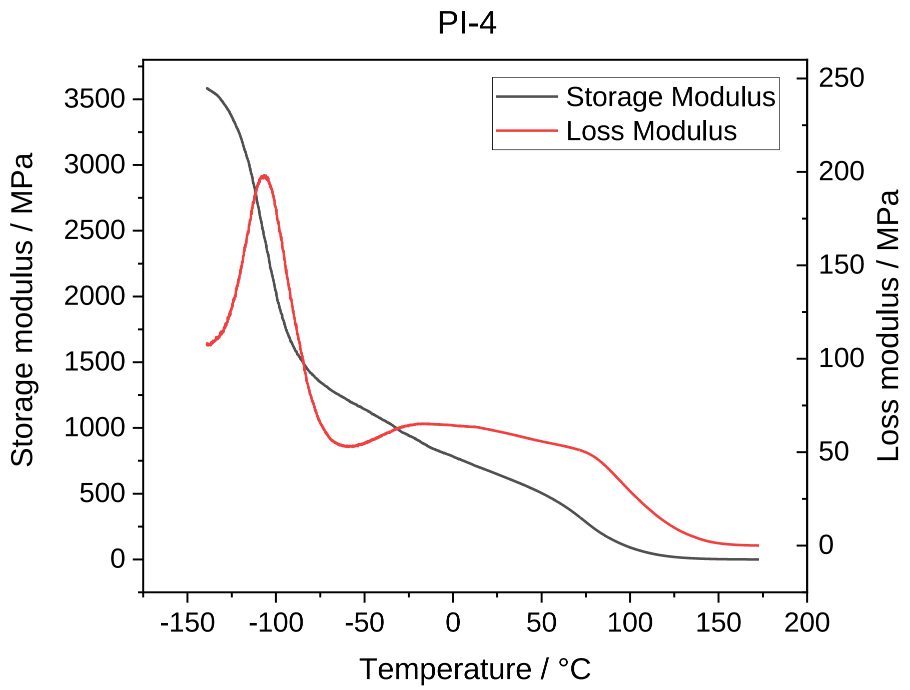

Figure S 8: Storage and loss modulus curves of ***PI-4.***

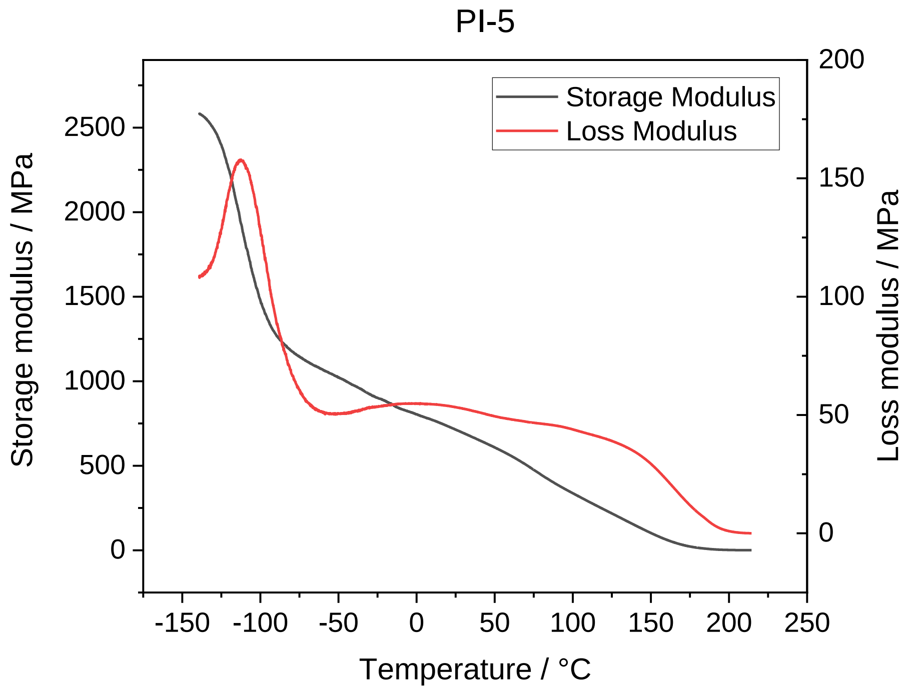

Figure S 9: Storage and loss modulus curves of ***PI-5.***

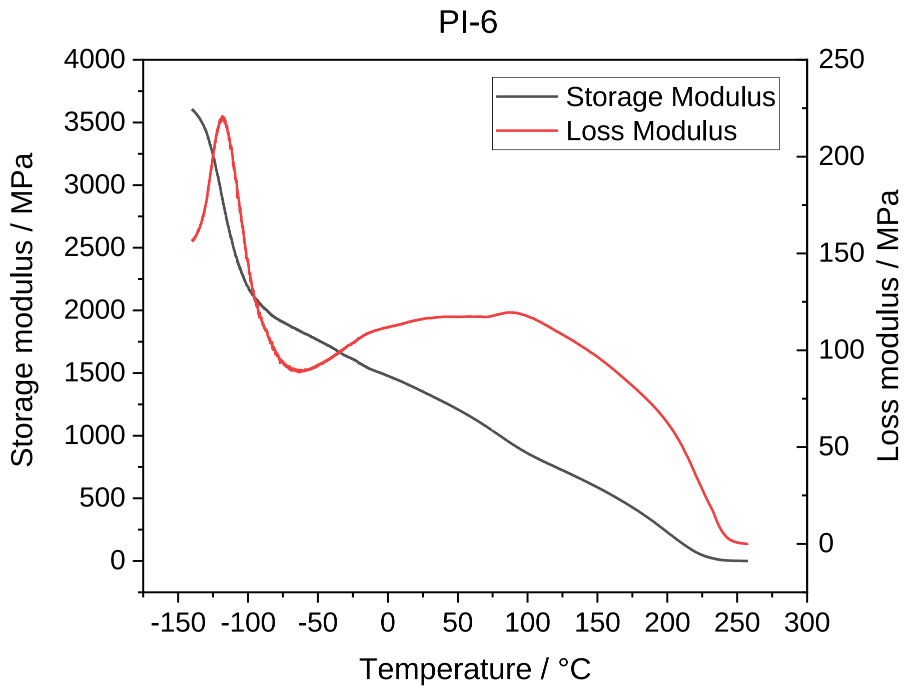

Figure S 10: Storage and loss modulus curves of ***PI-6.***

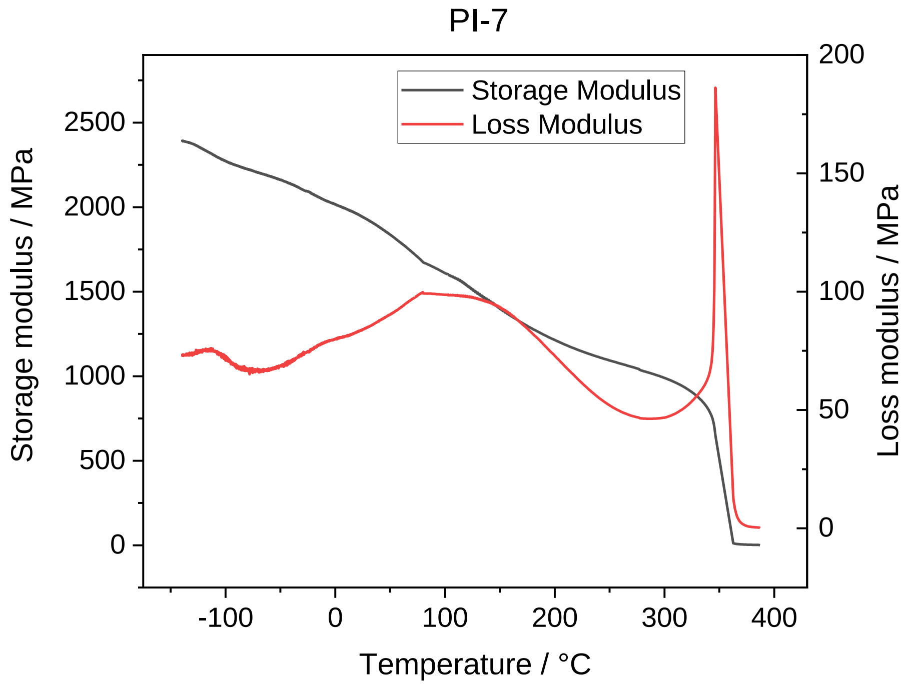

Figure S 11: Storage and loss modulus curves of ***PI-7.***

Stretchable electrodes

a)

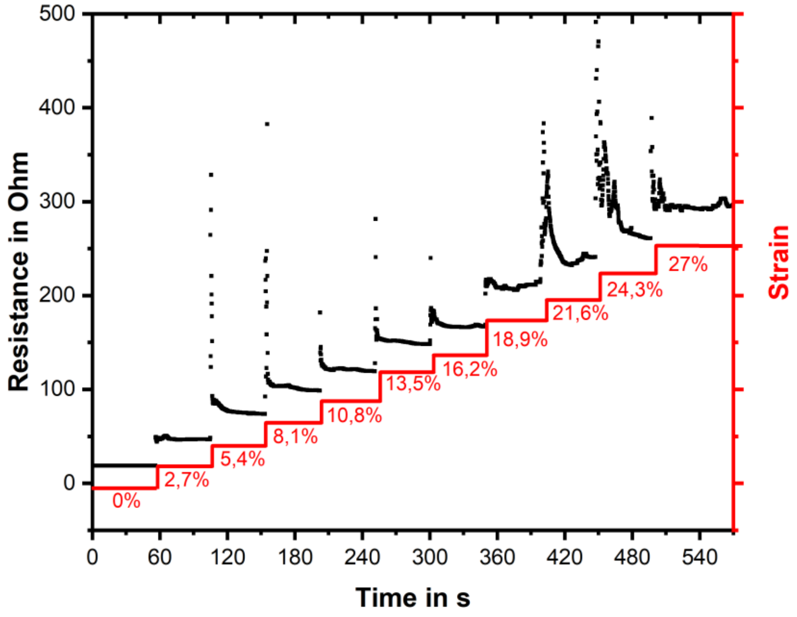

b)

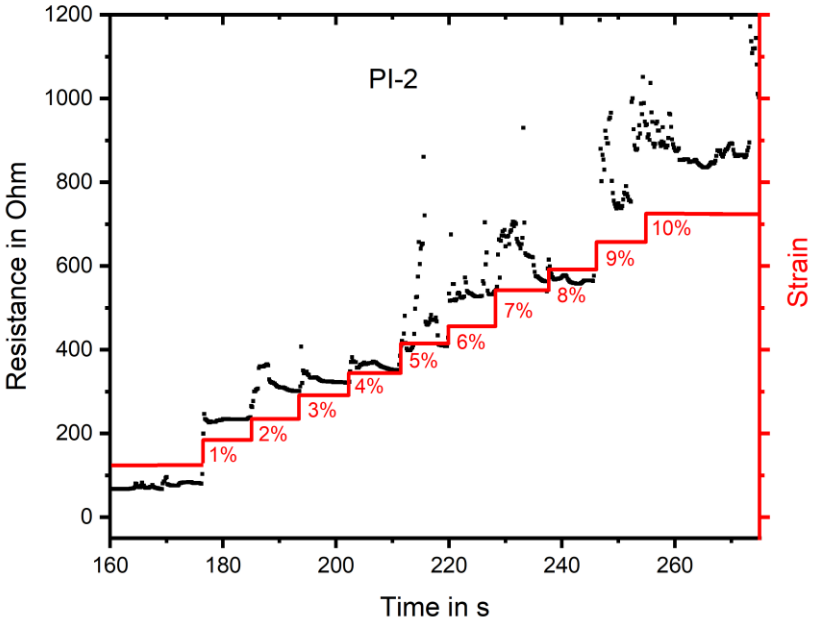

Figure S 12: Resistance (black, left) as a function of strain (red, right) for a stretchable thin-film electrode on a (a) ***PI-1*** *and (b)* ***PI-2*** *sample. The electrode was stepwise stretched up to a) 27% in 2,7% steps with 60 s rest time and 0,5 mm/s stretching speed and b) 10% in 1% steps respectively with a 10s rest time and a stretching rate of 0,1 mm/s. The stepwise increase in resistance closly follows the mechanical deformation. Spikes in resistance during stretching or immediately after a strtching step are due to the rigid-soft connections between multimeter and stretchable electrode.*

Dielectric elastomer actuator sample preparation test

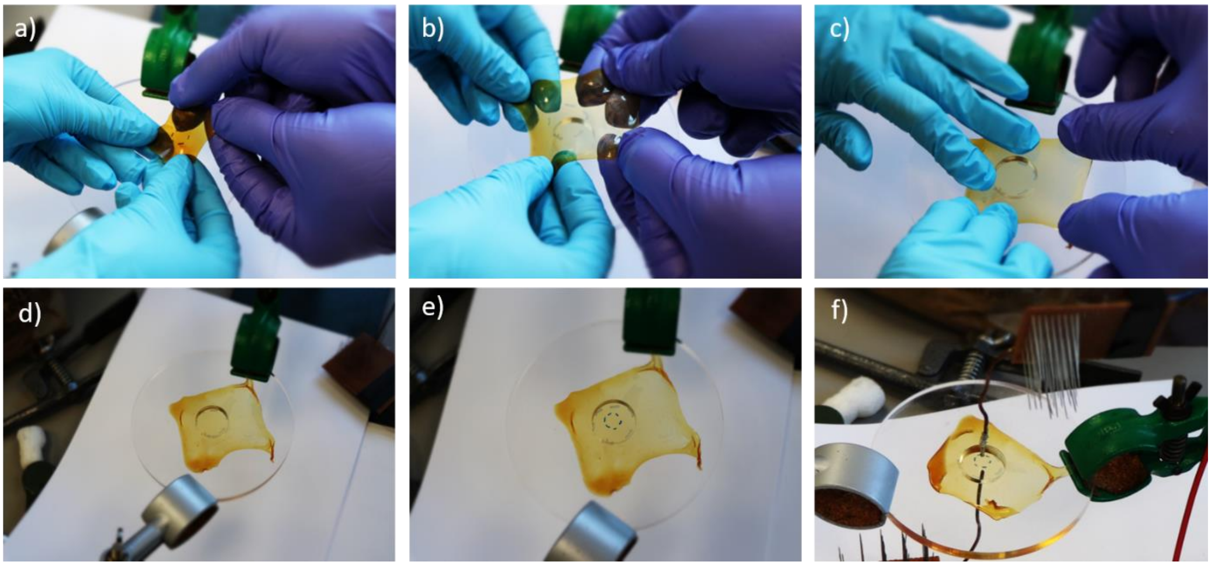

Figure S 13: Dielectric elastomer actuator setup: a) The elastomer membrane ist carefully radially prestretched to b) 135% with prestretch. The prestretch is controlled by means of a ring with a diameter of 8,5 mm which was manually drawn on the membrane. c) The membrane is attached to a PMMA disk with a 20 mm circular hole in the center so that it can be accessed from both sides. Adhesion of the membrane to PMMA is ensured by its own surface stickiness. d) The PMMA disk is mounted to allow accessing the membrane suspended over the hole. e) For reference a circle is drawn with permanent marker onto the membrane. f) Needle electrodes connected to a high voltage power supply are positioned close to the suspended membrane for actuation.

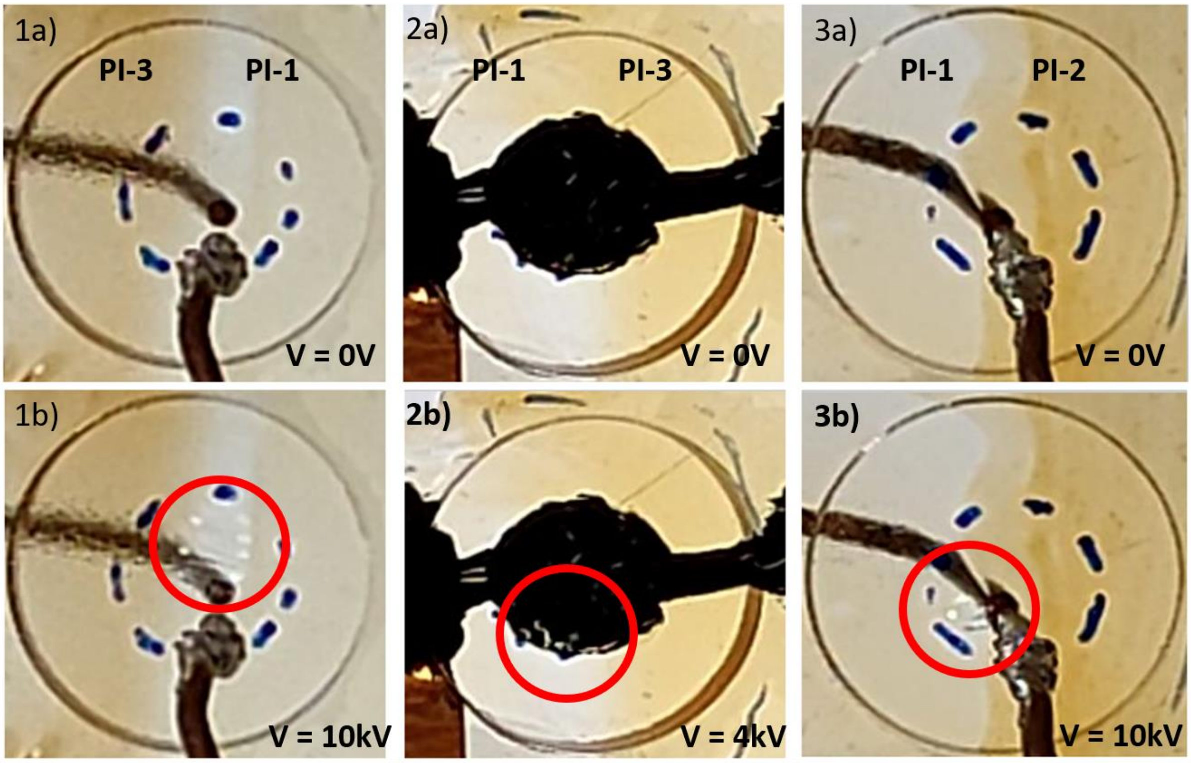

Figure S 14: (1-3) Graded elastomer tested as dielectric elastomer actuator: (1a) and (b) Gradient sample of ***PI-3*** *and* ***PI-1*** *at 0kV and 10kV respectively with actuation (marked with red circles)close to position of needles, (2a-b)* ***PI-1*** *and* ***PI-3*** *with stretchable electrode made from carbon grease at 0kV and 4kV respectively and (3a and b)* ***PI-1*** *and* ***PI-2*** *at 0kV and 13kV respectively. Actuation always was observed at the softest area of the sample.*

NMR spectra

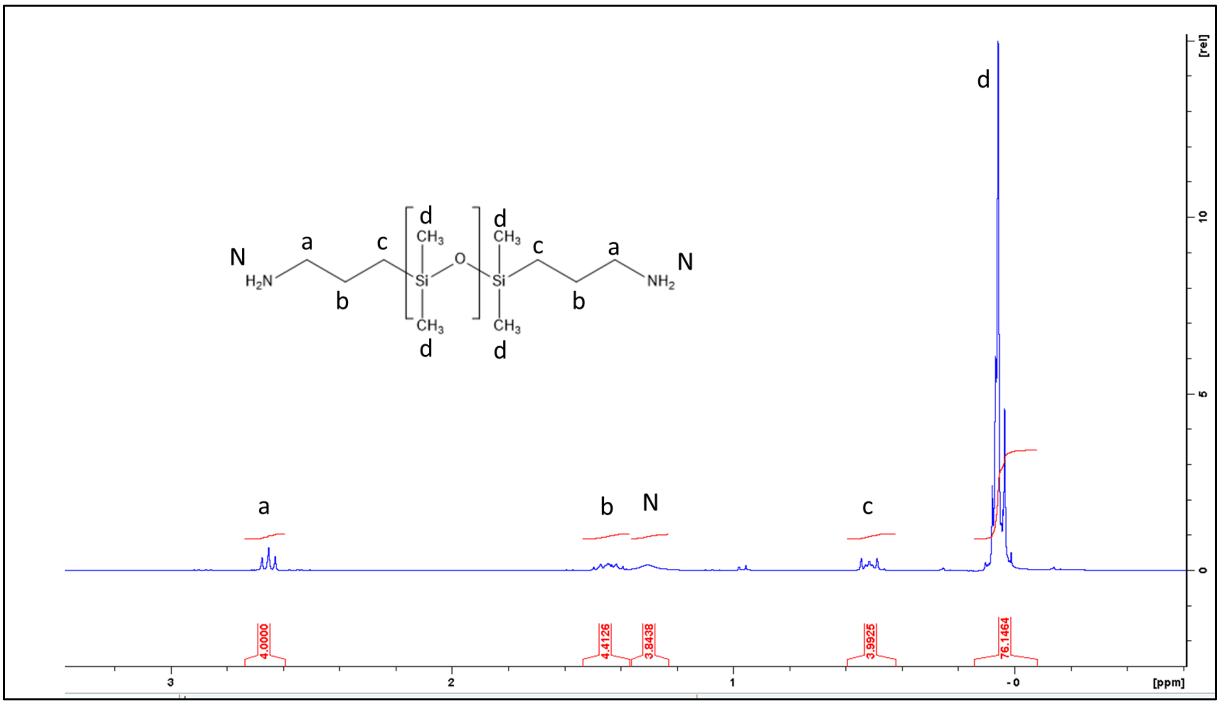

Figure S 15: ^{1}H NMR of PDMS A11, recorded in $CDCl_3$ at 300 MHz

<u>Dielectric measurements</u>

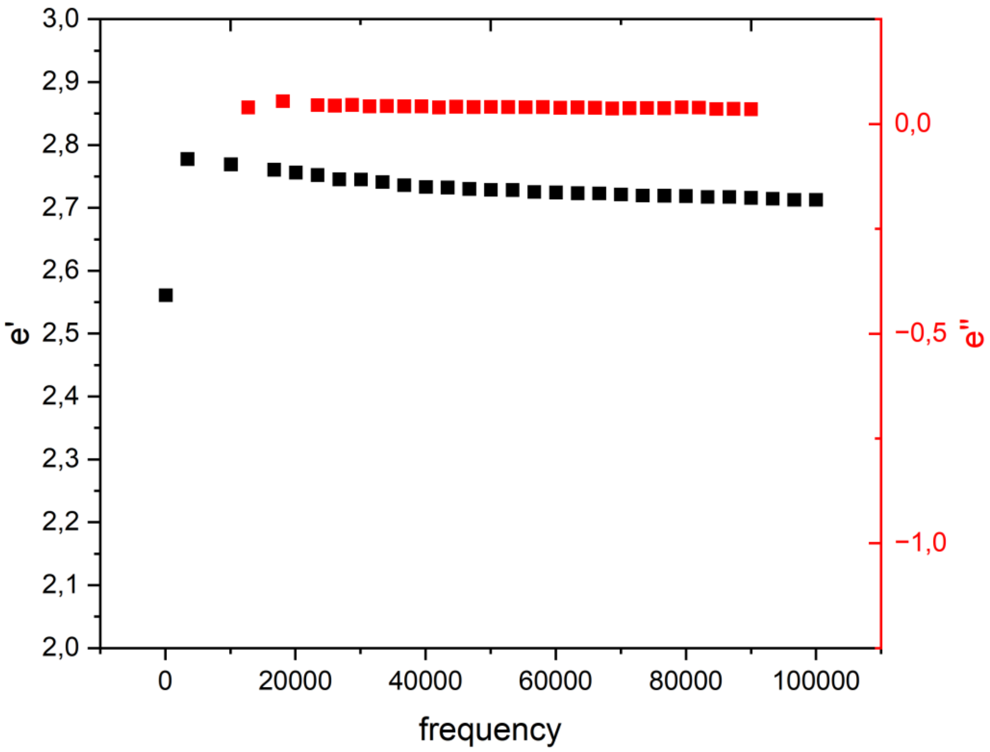

Figure S 16: e‘ and e‘‘ of PI-1 over a frequency range from 100 Hz to 100 kHz

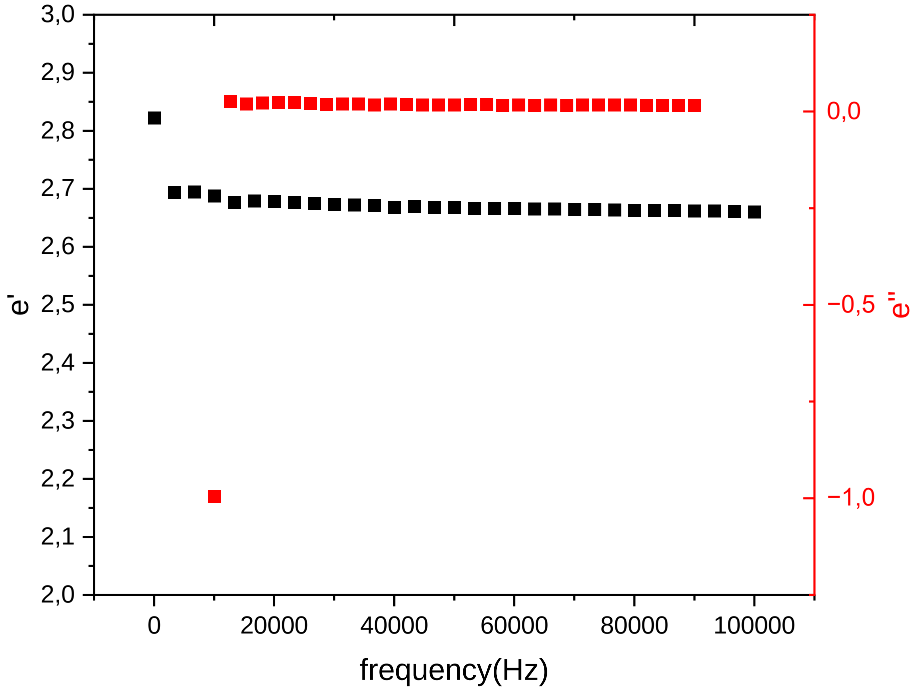

Figure S 17: e‘ and e‘‘ of PI-2 over a frequency range from 100 Hz to 100 kHz

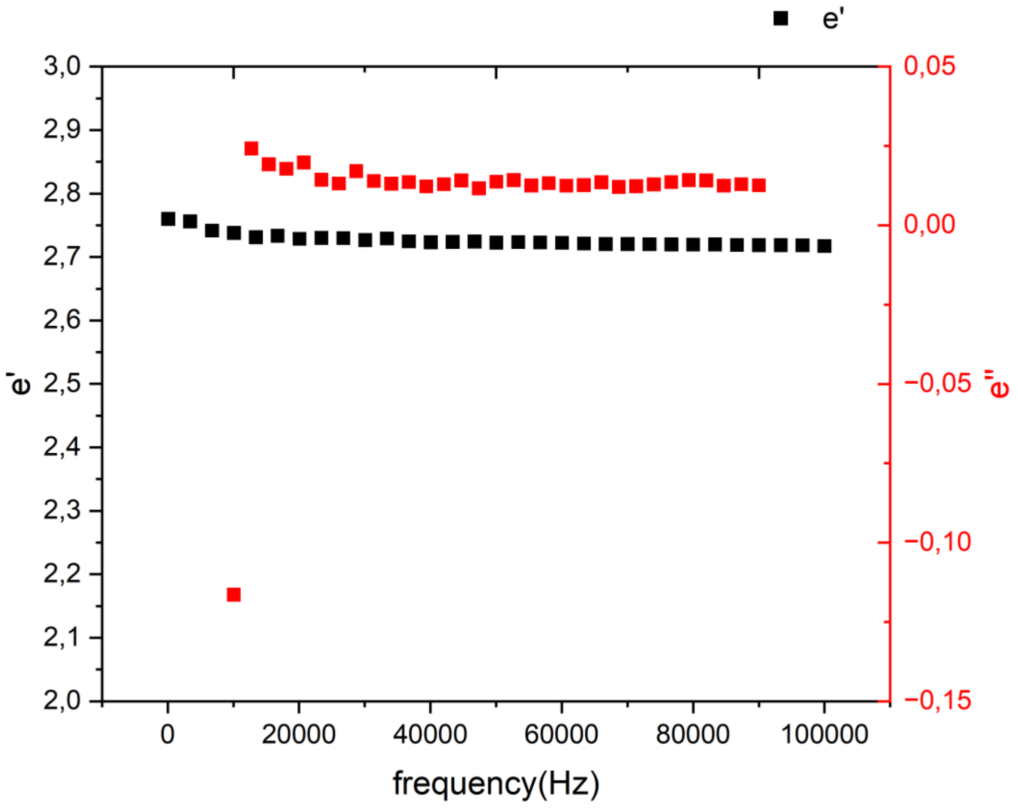

Figure S 18: e′ and e′′ of PI-3 over a frequency range from 100 Hz to 100 kHz

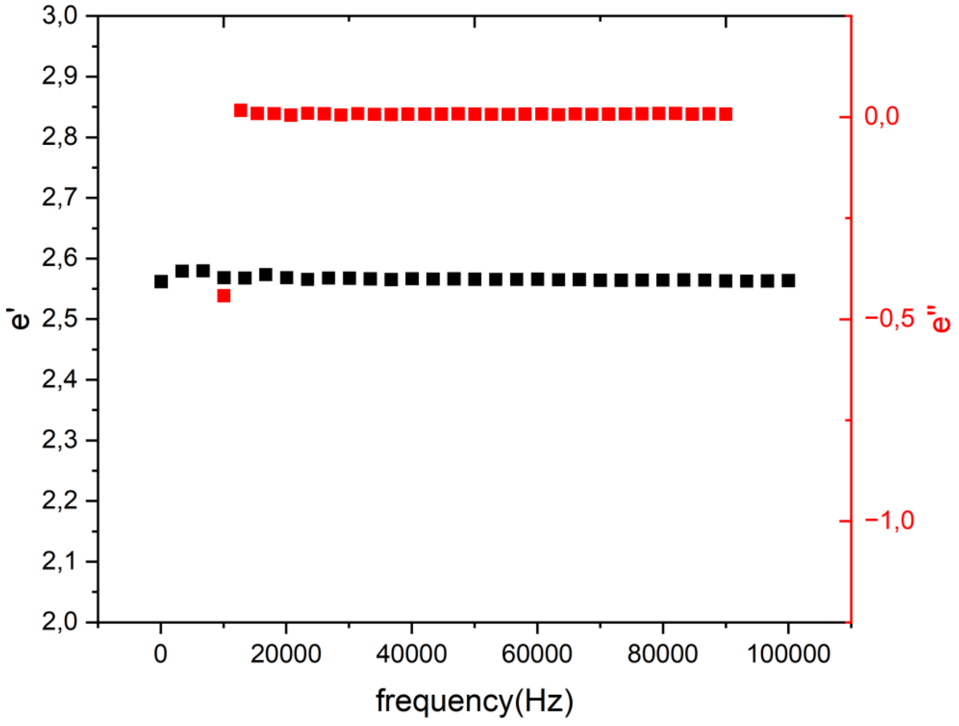

Figure S 19: e′ and e′′ of PI-4 over a frequency range from 100 Hz to 100 kHz

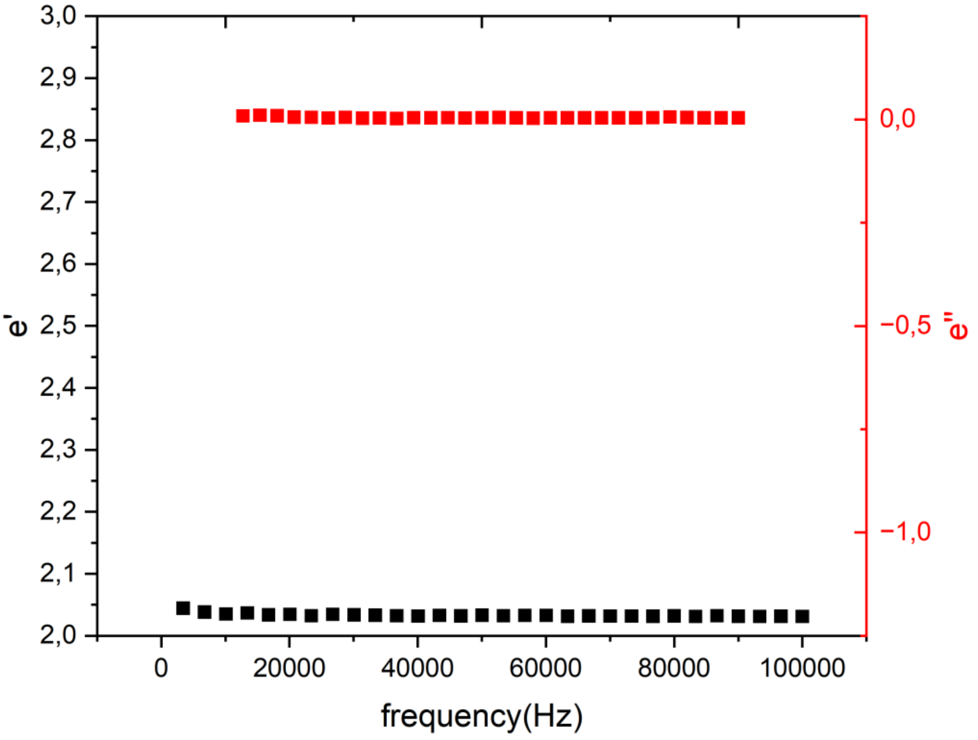

Figure S 20: e‘ and e‘‘ of PI-5 over a frequency range from 100 Hz to 100 kHz

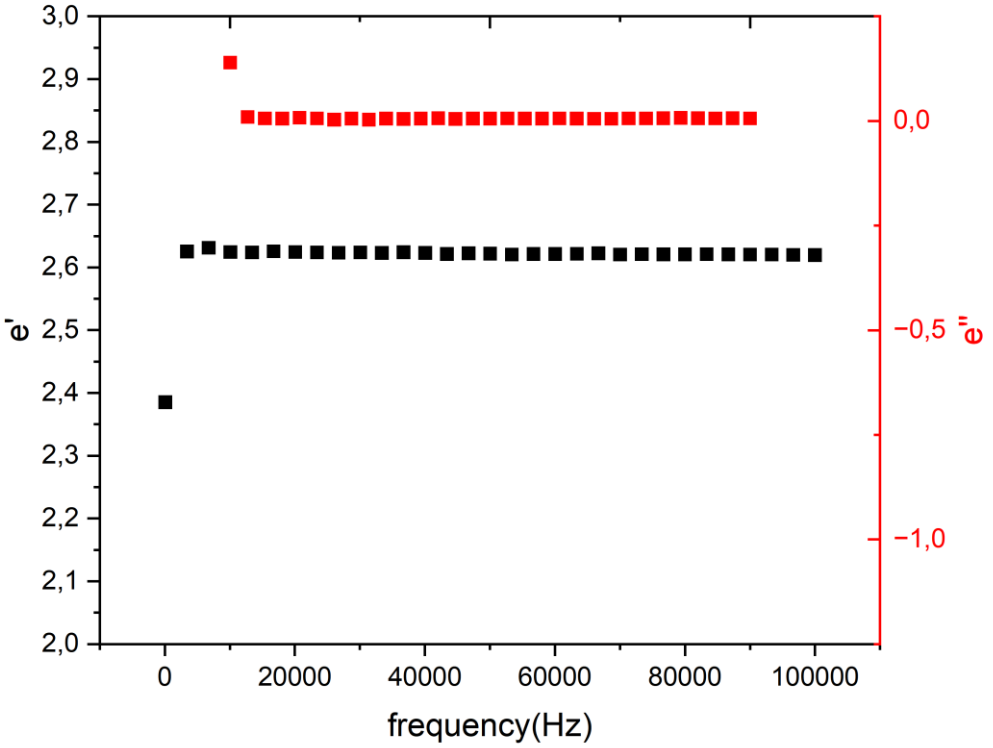

Figure S 21: e‘ and e‘‘ of PI-6 over a frequency range from 100 Hz to 100 kHz

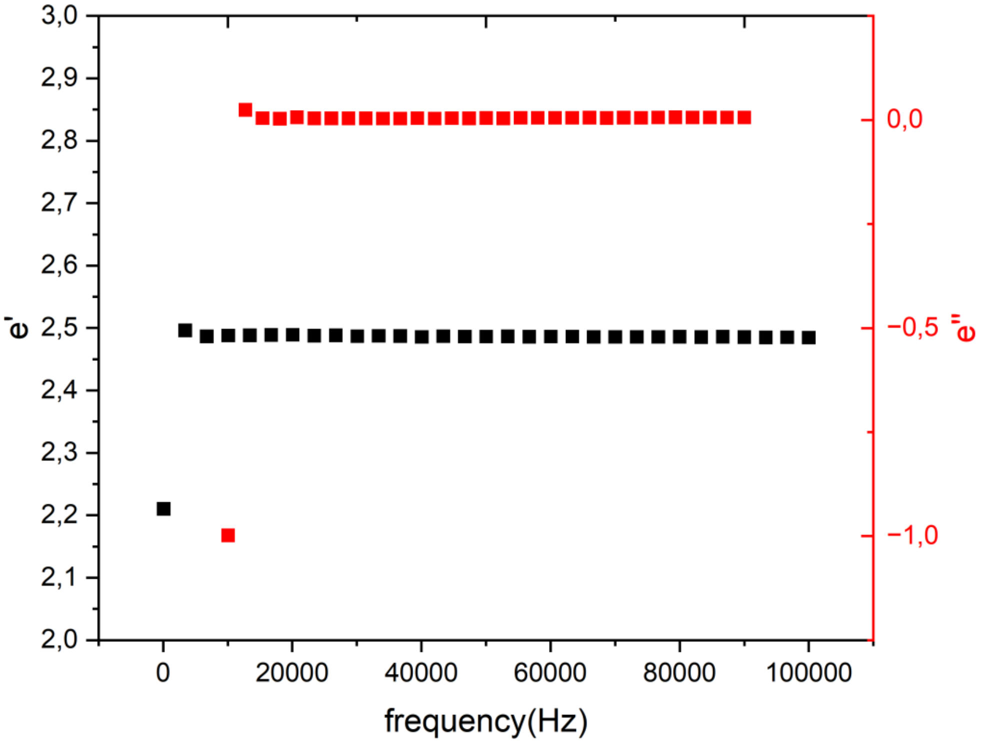

Figure S 22: e‘ and e‘‘ of PI-7 over a frequency range from 100 Hz to 100 kHz
